# Molecular Variants and Their Risks for Malignancy in Cytologically Indeterminate Thyroid Nodules

**DOI:** 10.1089/thy.2019.0278

**Published:** 2019-11-14

**Authors:** Whitney S. Goldner, Trevor E. Angell, Sallie Lou McAdoo, Joshua Babiarz, Peter M. Sadow, Fadi A. Nabhan, Christian Nasr, Richard T. Kloos

**Affiliations:** ^1^ Division of Diabetes, Endocrinology, and Metabolism, Department of Internal Medicine, University of Nebraska Medical Center, Omaha, Nebraska.; ^2^ Division of Endocrinology, Diabetes and Metabolism, Keck School of Medicine, University of Southern California, Los Angles, California.; ^3^ Veracyte, Inc., South San Francisco, California.; ^4^ Pathology Service, Massachusetts General Hospital, Boston, Massachusetts.; ^5^ Department of Pathology, Harvard Medical School, Boston, Massachusetts.; ^6^ Department of Otolaryngology, Massachusetts Eye and Ear, Boston, Massachusetts.; ^7^ Division of Endocrinology, Diabetes, and Metabolism, The Ohio State University Wexner Medical Center and Arthur G. James Cancer Center, Columbus, Ohio.; ^8^ Endocrinology and Metabolism Institute, Cleveland Clinic Foundation, Cleveland, Ohio.

**Keywords:** thyroid nodule, molecular testing, fine-needle aspiration, molecular diagnostics, indeterminate cytology, thyroid cancer

## Abstract

***Background:*** Gene panels are routinely used to assess predisposition to hereditary cancers by simultaneously testing multiple susceptibility genes and/or variants. More recently, genetic panels have been implemented as part of solid tumor malignancy testing assessing somatic alterations. One example is targeted variant panels for thyroid nodules that are not conclusively malignant or benign upon fine-needle aspiration (FNA). We systematically reviewed published studies from 2009 to 2018 that contained genetic data from preoperative FNA specimens on cytologically indeterminate thyroid nodules (ITNs) that subsequently underwent surgical resection. Pooled prevalence estimates per gene and variant, along with their respective positive predictive values (PPVs) for malignancy, were calculated.

***Summary:*** Our systematic search identified 540 studies that were supplemented by 18 studies from bibliographies or personal files. Sixty-one studies met all inclusion criteria and included >4600 ITNs. Overall, 26% of nodules contained at least 1 variant or fusion. However, half of them did not include details on the specific gene, variant, and/or complete fusion pair reported for inclusion toward PPV calculations. The PPVs of genomic alterations reported at least 10 times were limited to *BRAF^V600E^* (98%, 95% confidence interval [CI 96–99%]), *PAX8/PPARG* (55% [CI 34–78%]), *HRAS^Q61R^* (45% [CI 22–72%]), *BRAF^K601E^* (42% [CI 19–68%]), and *NRAS^Q61R^* (38% [CI 23–55%]). Excluding *BRAF^V600E^*, the pooled PPV for all other specified variants and fusions was 47%. Multiple variants within the same nodule were identified in ∼1% of ITN and carried a cumulative PPV of 77%.

***Conclusions:*** The chance that a genomic alteration predicts malignancy depends on the individual variant or fusion detected. Only five alterations were reported at least 10 times; *BRAF^V600E^* had a PPV of 98%, while the remaining four had individual PPVs ranging from 38% to 55%. The small sample size of most variants and fusion pairs found among ITNs, however, limits confidence in their individual PPV point estimates. Better specific reporting of genomic alterations with cytological category, histological subtype, and cancer staging would facilitate better understanding of cancer prediction, and the independent contribution of the genomic profile to prognosis.

## Introduction

The prediction of malignancy in thyroid nodules continues to evolve. Sonographic characteristics of thyroid nodules alone are not sufficient to predict the risk of malignancy for many nodules (1,2). Thyroid nodule fine-needle aspiration (FNA) is routinely performed to cytologically evaluate thyroid nodules that meet certain sonographic criteria (3,4). Management of nodules whose cytology is not clearly benign or malignant has been the most challenging. In the Bethesda System for Reporting Thyroid Cytopathology, the indeterminate categories of AUS/FLUS (atypia of undetermined significance [AUS] or follicular lesion of undetermined significance [FLUS]) and FN/SFN (follicular neoplasm [FN] or suspicious for a follicular neoplasm [SFN], including Hürthle cell [oncocytic] type) have an estimated risk of malignancy of 10–40% (5). Historically, these nodules commonly underwent repeat FNA and/or surgical removal. Approximately three-quarters of these were benign on surgical pathology, indicating the unnecessary surgical removal of many benign nodules. At the same time, malignant nodules potentially underwent inadequate initial treatment (6). Given the need for an improved means of predicting cancer risk and guiding surgical management in such nodules, novel diagnostic approaches have arisen, including the evaluation of genomic variants and fusions.

Initial studies included single genomic alterations, then small panels of several genes, and most recently numerous alterations among many genes (7–12). Despite discoveries of specific genetic variants and fusions in thyroid cancer specimens, many of these studies are not necessarily applicable to the preoperative evaluation of cytologically indeterminate thyroid nodules (ITNs) as they evaluated postsurgical histological rather than preoperative FNA specimens. Other studies only evaluated specific tumor histologies that are uncommon or differ strikingly from those seen among cytologically ITNs. Furthermore, it is often difficult to separate data specific to AUS/FLUS and FN/SFN nodules from those suspicious for malignancy (SFM), where the variant may have a different positive predictive value (PPV). For this reason, we examined the available published data on individual gene variants and fusions in preoperative cytologically indeterminate thyroid FNA samples from cohorts representative of routine clinical practice to determine their predictive values for thyroid malignancy.

## Review

### Literature search

**FIG. 1.**
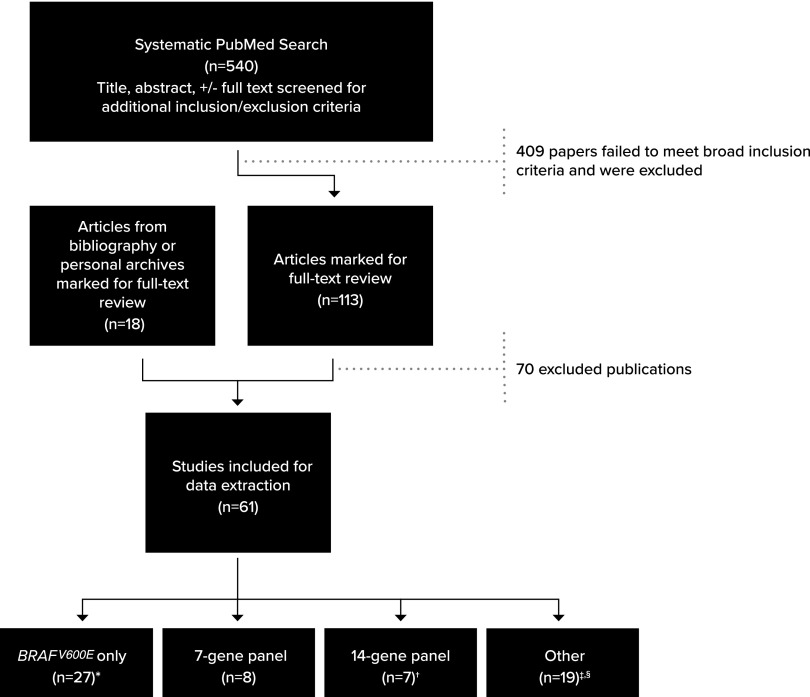
Literature review flowchart. Breakdown of search results and study inclusion. See Supplementary Table S2 for details on exclusion. *One reflexed to *RAS* if *BRAF* was negative (no *RAS* positives in indeterminate nodules) (30), while another only assessed *BRAF* for variants and *RET/PTC* fusions (one *RET/PTC1* was noted) (31). ^†^One study (32) combined data for patients run on either 7-gene or 14-gene panel. ^‡^Nodules from certain publications (33–36,38) were tested by 7- or 14-gene panels but only reported data on a subset of genes, variants, and/or fusions. ^§^One publication (37) analyzed nodules and reported data on two different panels.

To identify presurgical thyroid FNA specimens with AUS/FLUS or SFN/FN cytology that underwent molecular testing and resection, we performed a PubMed search for studies published between January 1, 2009, and December 31, 2018 (see Supplementary Data S1 for the search keywords and search parameters). The resulting 540 abstracts were reviewed, and 113 publications that potentially met the inclusion criteria described below were combined with an additional 18 publications (9,13–29) identified from bibliographies or personal files. These 131 publications underwent a detailed review and data extraction by at least 2 reviewers (Fig. 1). A second author independently repeated the original search and reviewed resulting abstracts identifying no additional publications that met our inclusion criteria.

### Inclusion/exclusion criteria

The reviewed literature included both US and international studies, but three studies (39–41) written in languages other than English were excluded. Information on each study was extracted for the categories below to ensure the nodule and its corresponding data came from a representative cohort of tested patients (i.e., presurgical FNA of nodules with AUS/FLUS or SFN/FN cytology). Examples of nonrepresentative cohorts/patients include: publications of unique patients, pediatric cases, or series that selectively included only some histopathologies (e.g., analysis limited to papillary thyroid cancer [PTC] nodules only). Full details of included and excluded publications are listed in Supplementary Tables S1 and S2, respectively.

- Presurgical sampling method:
  ○ Studies included molecular testing performed on multiple types of presurgical samples: dedicated FNAs, needle washings, core biopsy, slide smears, or slide scrapings. Those with molecular results only performed on postsurgical tissue were excluded.
  ○ Both prospective and retrospective studies were reviewed, but only studies pertaining to nodules with molecular testing and corresponding histological confirmation were included.
- Cytological category:
  ○ ITNs, defined here as The Bethesda System for Reporting Thyroid Cytology: (AUS/FLUS and/or SFN/FN) or other cytological equivalents (Thy3a, Thy3f, TIR3a, TIR3b, etc.), were included.
  ○ Cohorts referencing indeterminate cytology but not distinguishing between specific Bethesda categories had the possibility of including SFM samples. These studies were excluded unless data from the SFM samples could be separated from the molecular results of the other indeterminate specimens.
- Molecular techniques and gene inclusions:
  ○ All molecular laboratory techniques were included, unless the authors specifically reported that the technique had a high potential for unreliable detection (17,42,43).
  ○ The genes analyzed in each cohort, along with specific notation as to which genes and/or fusion pairs had positive results was recorded.

### Discrepancies and overlap

Extracted data were compared by at least two reviewers per publication. Discrepancies were resolved by rereview, discussion, and the involvement of an additional reviewer if necessary. To avoid the potential of including nodule data more than once, studies published from the same institution were evaluated for potential cohort overlap. Studies with apparent overlap were identified, and the largest and/or latest study was included. Additionally, unless a review article separately analyzed a novel patient cohort (34), review articles were excluded to minimize potential for overlap. Some patient overlap may remain due to limited descriptions of cohorts within the respective studies.

### Data extraction and PPV calculation

There was strong heterogeneity among the genes, variants, and fusions analyzed across the reviewed publications. The full list of variants and/or fusions assessed in most of the panels was not listed in the studies, so tracking of what was included in the panel was usually limited to the information provided for those nodules with genomic alterations. Information on any altered gene, along with the specific amino acid change, was collected when available. Data on the specific nucleotide change, however, were extremely limited, so all predictive data were analyzed at the level of the amino acid alteration for sequence variants. Both genes involved in a fusion pair were also documented, and samples with more than one variant and/or fusion in the same nodule were counted as positive for “multiple” variant status. When specific amino acid or fusion partner data were not available, the missing element was tracked as “unknown.”

Variant data from each included publication were summarized by the total number with that variant and total true positives (TP) and false positives (FP). This permitted a risk of malignancy [(TP/(TP+FP)] associated with that variant on a study-by-study level. All data from the same variant were then combined to create an overall variant risk of malignancy across all studies. Variants without the full designation were tallied separately at the gene, but unknown variant-level and similar study-specific and gene-specific risk of malignancies were generated. Significance of the difference between two independent proportions was calculated from the *z*-ratio and associated two-tailed probabilities (44).

### Study inclusion

Sixty-one of the final 131 publications met the inclusion criteria (Fig. 1; Supplementary Table S1). This review included 4648 presurgical nodules having Bethesda AUS/FLUS and/or SFN/FN cytology with corresponding histological outcomes.

The main geographic origin of included studies was United States (38%), Europe (29%), and South Korea (26%). Overall, 47% of publications analyzed only a single gene, most commonly those from South Korea (94%) compared with those from Europe (50%) or the United States (9%). The only publications to report panels of >7 genes were from the United States (52% of U.S. studies).

### Sampling

Eighty-six percent (*n* = 4021 nodules) of studies analyzed data from dedicated FNA samples, whereas 12% analyzed samples (*n* = 566) from scrapings off cytology slides. Only one study (45), evaluating *NRAS*, used core biopsy to obtain samples (*n* = 61). Regardless of sampling method, 80% of studies had data from both indeterminate categories (i.e., AUS/FLUS and SFN/FN).

### Panels used

Twenty-seven (44%) studies focused exclusively on analysis of *BRAF^V600E^*. Sixteen other different gene/fusion combinations were assessed across the remaining studies, including 8 analyzing a panel of 4 genes and 3 fusions (7-gene panel), and 6 studies examining an expanded 14-gene panel. Although these 14-gene panels included the same genes assessed for sequence variants, the fusions analyzed varied and the full fusion set was not always listed. The remaining 20 studies looked at other combinations of genes/fusions/panels ranging from 1 to 524 genes (Fig. 1; Supplementary Table S1; Supplementary Data S2). Some publications involving larger gene panels only reported data on a subset of genes/variants/fusions (e.g., tested samples by full 14-gene panel but only reported on *TSHR* findings) (33–36,38), limiting the interpretation of the full panel.

### Cumulative molecular results

**FIG. 2.**
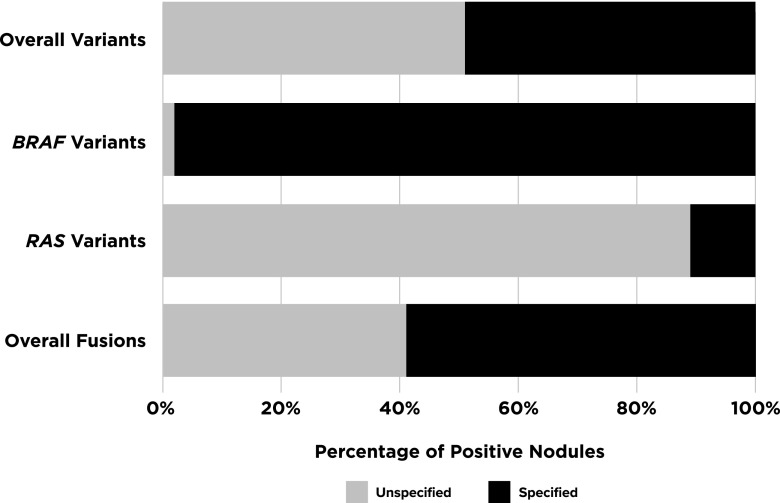
Specified breakdown of variants/fusions. Percentage of positive nodules identified with unspecified amino acid changes or incomplete fusions pairs versus those that had them specified. *H-/K-/N-RAS* data are significantly lower for specified variant designation than *BRAF* (*p* < 0.0001) when looking at these data for sequence variants alone.

Of the 4648 total nodules, 1187 (25.5%) were positive for at least 1 variant or fusion, but half did not include the complete fusion pair and/or the specific amino acid change (Fig. 2). Of those with a known, specified single alteration, 94% were sequence variants and 6% were fusions. Taking together both known single sequence variants and fusions, overall PPV was 47% or 86% (*p* < 0.0002) depending on whether *BRAF^V600E^* was removed or included, respectively. Twenty-two additional nodules from 10 studies had more than 1 variant. Table 1 details the various alterations reported.

**Table 1.** Reported Variants and Fusions

*Gene*	*Amino acid change*	*TP/total positives*	*PPV*	*References*
Sequence variants				
*BRAF*	V600_K601>E	1/1		(46)
***BRAF***	**V600E**	**422/430**	**98.1%**	(16,18,20,22,24,25,28,30–32,46,49–53,55,56,59,61,62,64,66–88)
*BRAF*	Unknown	9/9	—	(32,37,47,48)
***BRAF***	**K601E**	**5/12**	**41.7%**	(46,49–55)
*EIF1AX*	A113 splice	0/4		(53)
*EIF1AX*	Unknown	3/9	—	(32,37,48,49,55)
*FAT1*	V912I	0/1		(9)
*HRAS*	Unknown	79/104	—	(34,35,37,46–51,55,56)
*HRAS*	G12V	1/2		(33)
*HRAS*	Q61H	1/1		(57)
*HRAS*	Q61K	3/3		(55,57)
*HRAS*	Q61P	2/2		(57)
***HRAS***	**Q61R**	**5/11**	**45.4%**	(33,53,55,57–59)
*KRAS*	Unknown	32/58	—	(34,35,37,46,47,49–51,55,56,60)
*KRAS*	G12C	0/2		(55,61)
*KRAS*	G12D	0/2		(55)
*KRAS*	G12V	0/2		(55,59)
*KRAS*	Q61R	2/2		(59,61)
*MET*^a^	Unknown	3/4		(37,56)
*NRAS*	Unknown	202/296	—	(34,35,37,45–52,55,56,60–63)
*NRAS*	Q61K	3/7		(57–59,61,64)
***NRAS***	**Q61R**	**12/32**	**37.5%**	(33,53,57–59,61)
*PTEN*	Unknown	0/2	—	(49,63)
*RAS* (not otherwise specified)	Unknown	61/83	—	(65–70)
*RET*	Unknown	1/1	—	(32)
*TERT*	Unknown promoter	1/2^b^	—	(48)
*TERT*	C250T	1/1		(46)
*TERT*	C228T	6/6		(46,50,54,69)
*TP53*	Unknown	0/1	—	(32)
*TSHR*	I630L	1/1		(38)
*TSHR*	D633H	0/1		(38)
*TSHR*	I486F	0/1		(38)
*TSHR*	T632A	0/1		(38)
*TSHR*	P631L	0/1		(38)
*TSHR*	I586F	0/1		(38)
*TSHR*	L512Q	0/1		(38)
*TSHR*	L512R	0/1		(38)
*TSHR*	M453T	0/3		(9,36)
*TSHR*	D633E	0/1		(38)
*TSHR*	I486M	0/1		(36)
*TSHR*	I568T	0/1		(36)
*TSHR*	Unknown	1/7	—	(37,48,50,55)
Fusion pairs				
*SND1_BRAF*	n/a	0/1		(9)
*ETV6_NTRK3*	n/a	1/1		(53)
***PAX8_PPARG***	**n/a**	**11/20**	**55.0%**	(9,32,47,48,53,55,56,58,65,67,68)
*RET_PTC1*	n/a	3/3		(31,53,62)
*RET_PTC3*	n/a	2/2		(47)
*RET_PTC* (unknown)	n/a	2/2	—	(66,67)
*THADA_IGF2BP3*	n/a	5/5		(48,53,55)
*THADA_*(unknown)	n/a	8/9	—	(49,50,63)
(unknown)_*ALK*	n/a	1/1	—	(49)
(unknown)_*NTRK1*	n/a	1/1	—	(49)
(unknown)_*NTRK3*	n/a	3/3	—	(49,50)
(unknown)_*PPARG*	n/a	5/6	—	(37,49,50)
Multiple mutations				
*TP53* (T221I) and *TP53* (Q331X)		0/1		(53)
*EIF1AX* and *TSHR*		0/1		(55)
*HRAS* and *RET*		0/1		(32)
*NRAS* (Q61R) and *RET/PTC1*		1/1		(55)
*NRAS* and *TSHR*		1/1		(32)
*NRAS* and *PIK3CA* and *TP53*		1/1		(50)
*TERT* (C250T) and *BRAF* (K601E)		1/1		(46)
*TERT* (C228T) and *BRAF* (K601E)		1/1		(54)
*TERT* (C228T) and *KRAS* (codon 12)		1/1		(46)
*TERT* (C228T) and *NRAS* (codon 61)		3/3		(46)
*TERT* (C228T) and *NRAS*		2/2		(50)
*TERT* and *EIF1AX* and *NRAS* (Q61K)		1/1		(55)
*TERT* and *BRAF* and *AKT1* and *PIK3CA*		1/1		(32)
*NRAS* and *TSHR* and *TERT*		1/1		(63)
*NRAS* and *TERT*		1/1		(63)
*HRAS* and *EIF1AX*		1/2		(37,63)
*NRAS* and *TP53*		1/1		(56)
*GNAS (Q227H)* and *EIF1AX (R13P)*		0/1		(36)

List of all reported sequence variants and fusions (whether amino acid change is specified or unspecified) and the corresponding PPV and publications. Bold entries were present with amino acid change in ≥10 nodules, which was our threshold for reporting PPV or TP over total positives. Only 5 of 36 reported variants were reported with this frequency.

^a^ Although one study (37) specifically referred to mutation in *MET*, panel did not mention *MET* as a gene being analyzed for variants. The other study (56) did not say how *MET* was affected, only that it was positive.

^b^ The TP in this group was noted as a TERT promoter variant, but no specific amino acid change was listed.

n/a, not applicable; PPV, positive predictive value; TP, true positive.

### Sequence variants

Only 12 genes had a variant identified, but of those reported as positive, 52% did not list the specific amino acid change (Fig. 2). *BRAF^V600E^* was the most commonly analyzed variant (54 of 61 studies), with half of these publications assessing only for this variant. *NRAS^Q61R^*, *HRAS^Q61R^*, and *BRAF^K601E^* were the only other specified sequence changes to be found in more than 10 nodules each. These sequence-change variants, along with *BRAF^V600E^*, made up 76% of the total known sequence variant positive nodules in multi-gene analyses. Twenty-six additional variants across 8 genes were noted in 50 nodules.

For all variants with known sequence changes, the overall PPV was 87% (individual variant PPV ranged from 0% to 100%). *BRAF^V600E^*, *NRAS^Q61R^*, *HRAS^Q61R^*, and *BRAF^K601E^* accounted for 95% of all TP and have a combined PPV of 91%, but the high PPV is primarily driven by *BRAF^V600E^*. When *BRAF^V600E^* is removed, the cumulative PPV of the remaining 3 variants was lowered significantly to 40% (*p* < 0.0002).

#### BRAF^V600E^

Fifty-four of 61 studies assessed samples for *BRAF^V600E^*, 9 of which did not have any nodules positive for this variant (Supplementary Fig. S1). In these 54 publications, 430 of 4293 nodules (10%) were V600E positive. Histology was malignant in 422, corresponding to a PPV of 98% (95% confidence interval [CI 96–99%]).

#### BRAF^V601E^

The K601E variant for *BRAF* was the third most reported known variant with 12 nodules reported across 8 studies. Five TP were reported across 4 studies, yielding a PPV of 42% [CI 19–68%].

#### RAS

**FIG. 3.**
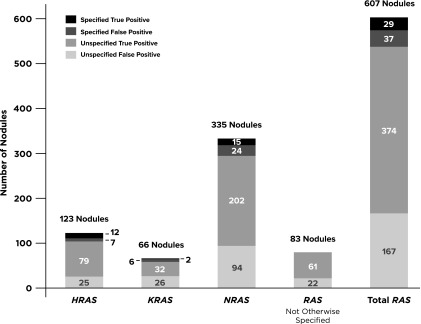
*RAS* data breakdown. Ratios of specified versus unspecified amino acid changes separated by data for all *RAS*-positive nodules, data for *RAS* without the gene specified, and data for *H-/K-/N-RAS* along with the corresponding breakdown of true and false positives. This figure shows that specified data were very minimal for this gene category (66/607 *RAS*-positive nodules).

*RAS* genes were assessed in some manner in 94% of publications that analyzed more than just *BRAF^V600E^*. Across these 33 publications, 607 of 2674 nodules (23%) contained a *RAS* variant with a corresponding 66% PPV [CI 63–70%]. However, *RAS* gene and/or variant specification was missing from 541 (89%) of these nodules (Figs. 2 and 3). When considering only the remaining 66 nodules from 8 studies, the PPV was reduced to 44% ([CI 33–56%], *p* = 0.0003). When the specific *RAS* gene was provided, *NRAS* was 1.8 times more likely to be altered than the other 2 genes combined (*n* = 335 vs. 123 *HRAS* + 66 *KRAS*). The gene-specific data showed differing PPV across *RAS* genes when variants were specified (*HRAS* 63%, *NRAS* 38%, *KRAS* 25%) versus when unknown variants were also included (*HRAS* 74%, *NRAS* 65%, *KRAS* 51%).

*NRAS^Q61R^* was the most reported *RAS* variant and had malignant histology in 12 of 32 nodules (PPV = 37.5% [CI 23–55%]). It was the second most reported specific variant after *BRAF^V600E^*, but with significantly lower PPV (*p* < 0.0002). The fourth most reported variant was also in the *RAS* family, *HRAS^Q61R^*. It was present in 11 nodules across 6 studies with a PPV of 45% [CI 22–72%].

#### *TERT* promoter

Fifteen studies (25%) assessed for *TERT* promoter (*TERT*p) (C228T and C250T) variants. Overall, 1.6% of nodules in these studies contained a specified *TERT*p variant, either as a solitary variant (43%) or as a part of a multiple variant (57%). Solitary *TERT*p variants were found in 9 nodules across 5 publications with a PPV of 88% [CI 56–98%], although the specific *TERT*p was not always specified. When the specific promoter variant was noted (7 of 9 cases), C228T was more frequently reported than C250T (6 nodules and 1 nodule, respectively).

### Fusions

Fusions were reported in 22 of the 61 studies (36%). Fifty-four nodules had fusions involving 13 genes with both known (59%) and unknown (41%) partners (i.e., not listing both partners in the fusion pair) (Fig. 2). Among studies that assessed fusions, 3% of nodules were positive for a single fusion; however, the total positives for any completely specified fusions were usually too small (*n* < 10) to confidently estimate a PPV point value. Overall, the PPV for fusions was 69% or 78% depending on whether both partners were or were not specified, respectively (range 0–100%, *p* = 0.3539).

#### PAX8/PPARG

Twenty nodules across 11 studies carried this fusion pair and accounted for 37% of all reported fusions and 63% of fusions in which both fusion partners are known. This fusion demonstrated a PPV of 55% [CI 34–78%]. An additional 6 nodules from 3 studies were noted as having a *PPARG* fusion (PPV = 83%), but the gene partner was not listed.

#### *RET*/*PTC*

Only 7 *RET*/*PTC* fusions were identified, 3 *RET/PTC1*, 2 *RET/PTC3*, and 2 with an unreported PTC partner, comprising 13% of all fusions. It was the second most noted fusion pair, although with a higher PPV of 100% [CI 57–100%].

#### *THADA* fusions

The *THADA/IGF2BP3* fusion was the third most reported fusion, reported 5 times across 3 studies with a cumulative PPV of 100% [CI 57–100%]. Nine additional nodules having *THADA* fusions without listing the corresponding fusion partner were noted in 3 publications, 1 being FP.

### Multiple variants in one nodule

Ten studies (approximately one-third of studies analyzing ≥1 gene) reported finding multiple variants in the same nodule with corresponding histological confirmation. Overall nodules with multiple variants comprised <1% of all nodules across these cohorts. Sixteen different combinations were seen with a cumulative PPV of 77% [CI 57–90%]. The various combinations, ranging from 2 different sequence variants in 1 gene to 4 variants in 4 separate genes, are listed in Table 1. Most combinations were unique or missing detailed nomenclature, making reliable point estimates of PPV for each combination difficult.

## Discussion

We evaluated the incidence and PPV of genetic variants and fusions on preoperative clinical specimens from >4600 thyroid nodules with indeterminate cytology from 61 publications. Our analysis was restricted to cohorts representative of general clinical practice (i.e., those with preoperative collection, indeterminate cytopathology, histological confirmation) so that we could understand the PPV of these genetic changes in similar cohorts.

Overall, 26% of nodules were positive for at least one variant and/or fusion. Sequence-changing variants made up the majority (94%) of aberrations found. However, approximately half of these cases only had information on genes involved and not the specific amino acid or resultant protein change, hence only a gene-level PPV could be calculated. This is important because it is likely that variants in the same gene are associated with different PPVs. This was particularly common within the *RAS* gene family (*HRAS*, *KRAS*, and *NRAS*). Unlike the 98% proper designation for *BRAF* variants, nodules positive for *RAS* variants were missing the specific gene or variant designation in 14% and 75% of cases, respectively (Fig. 3). Similarly, missing fusion partners limited our ability to reliably estimate predictive values for many specific fusion pairs.

American Thyroid Association (ATA) guidelines identify *BRAF^V600E^*, *RET/PTC*, and *PAX8/PPARG* as having high enough PPV (>95%) to be considered “rule-in” tests. Our data show a similarly high PPV for *BRAF^V600E^* (98%) and combined *RET/PTC* fusions (100%), but a much lower PPV (55%) for *PAX8/PPARG* fusions. The small individual sample size of the remaining reported variants in the literature creates PPVs with wide CIs. Outside *BRAF^V600E^*, the likelihood of cancer for those variants documented as positive in at least 10 nodules in the included studies (*BRAF^K601E^*, *HRAS^q61R^*, *NRAS^q61R^*, and *PAX8/PPARG* fusion) ranged from 37% to 55%. Multiple variants in the same nodule were rare, with an incidence of <1% in studies that assessed more than one gene and yielded a cumulative PPV of 77%. Although these risks are increased above the *a priori* cytological risk, these values are not high enough to consider these as “rule-in” results for thyroid carcinoma [i.e., ≥98.6% per ATA guidelines (3)].

### Importance of adequate sample size

Despite some panels having up to 524 genes, only 26 genes harbored a variant or fusion (Table 1), reflective of the low frequency of somatic variants observed in thyroid cancer surgical tissues compared with other cancers (7,89). Thirty-six separate sequence variants or fusions were noted, yet 44% were reported only once. Only 14% of specific variants or fusions (*n* = 5) were seen in ≥10 nodules with associated surgical histopathology. Without adequate sample sizes, confident point estimate calculations of PPV are not possible. An estimated sample size of close to 100 nodules affected with any given variant is needed to achieve a CI of ±10% allowing for more confident PPV estimates. Only *BRAF^V600E^* has been reported frequently enough among ITNs to meet this qualification. If the specific variant data had been documented for all *RAS*-positive nodules, better estimates of their true PPV may have been possible.

### Importance of assessing PPV by individual variant

An overall PPV of 68% was seen for all single variants/fusions positive by multi-gene panels. As in other cell types, thyroid nodules harbor a variety of genomic aberrations that have varying levels of association with cancer. Due to its markedly high specificity compared with most other variants, the frequency of *BRAF^V600E^* within a cohort could significantly affect the apparent cumulative PPV of the panel. Indeed, removing *BRAF^V600E^* data from the multiple panel studies reduced the overall remaining PPV to 49% (*p* = 0.0002).

It is well accepted that *BRAF^V600E^* has a much higher PPV than K601E, but corollary differences are less recognized for *RAS* and other variants. The data presented here suggest that variants in *KRAS* have a significantly lower PPV than variants in *NRAS* (*p* = 0.002), and as more variant-specific PPV data on *RAS* become available, this may also hold true at the variant level. Differing cancer risks among specific variants may contribute to the heterogeneity reported for *RAS* performance across different studies (90,91). Thus, assigning a risk interpretation to a panel or group of genes/variants rather than to individual specific variants may be less accurate. We also believe that it is likely that understanding tumor prediction and prognostics at the specific variant level will increase personalized prediction accuracy and treatment decisions (92).

### Limitations

Our goal was to include all relevant publications. There are known limitations for online searches, so we extended our search to include bibliographies of articles identified by the online search and personal libraries known to include articles relevant to this topic. To ensure the additional off-line search methods did not skew the results, data were reanalyzed using only the publications found via the online search and yielded no significant differences.

Additionally, the panel heterogeneity limited the ability to directly compare results of any two publications and estimate accurate incidences. This heterogeneity, along with data only available on operated positive cases in most studies, also made calculations of sensitivity unreliable.

Another potential limitation is that our analysis utilized local, largely unblinded histological diagnoses, as opposed to a blinded panel of expert histopathologists. Caution should be exercised in generalizing any single center's experience to other populations. Imperfect diagnostic concordance among pathologists is known, especially among follicular and oncocytic lesions, and tendencies to categorize such lesions as benign or malignant along with the unblinded nature of such diagnoses could impact locally derived PPVs and would generate heterogeneity among PPV estimates between institutions with differing tendencies (91).

We were concerned about generalizing PPV estimates on variants with lower specificities across multiple cytological categories having a wide variation in malignancy prevalence. There is a more similar pretest risk of malignancy across the AUS/FLUS and FN/SFN categories that do not extend to the SFM group. ATA guideline recommendations 17a and 20 suggest that SFM nodules be treated as if they were cytologically malignant nodules (3). The higher pretest risk of malignancy and the predominance of PTCs in higher categories would markedly influence a variant's PPV. It is for these reasons that our protocol and search terms were designed to capture data on AUS/FLUS and FN/SFN only. Future investigation into predictive values and clinical utility of these variants across other Bethesda cytological categories may be warranted.

Finally, our study is unable to fully quantify the impact of the noninvasive follicular thyroid neoplasms with papillary-like nuclear features (NIFTP) histological category upon PPV calculations. NIFTP are considered to have a low risk of malignant behavior following surgical excision and are considered as a cancer *in situ*. Most publications included in our study accrued patients before the formal recognition of NIFTP, and these neoplasms would have been labeled follicular variant of PTCs and considered malignant. Of the publications in our analysis that reported NIFTP histology in their results (22,27,37,46,48,53,54,63,69), we considered them as “malignant” for statistical purposes, consistent with the current desire that they undergo surgical resection as opposed to *in situ* observation. Thus, PPVs in our study estimate the combined probably of cancer or NIFTP. However, if NIFTP cases were to be included with the benign diagnoses, PPVs of variants found in these samples would be further lowered, particularly *THADA/IGF2BP3* fusions, which would be reduced from 100% to 20%.

### Reporting variants in future studies

Future studies with complete data could provide data to allow for (i) incidence and PPV refinement by variant and/or cytology subcategory, (ii) better correlation of certain variants to neoplastic and oncogenic subtypes (e.g., NIFTP) allowing for a more detailed risk prediction, and (iii) investigation of the independent contribution of the genomic profile to prognosis. We suggest a data chart (Supplementary Table S3) to standardize reporting in future studies.

While our study highlights the limited data available on the association of most variants and fusions to predict cancer among cytologically ITNs, data are beginning to emerge linking genomic alterations with specific types of neoplasms, their behavior, routes of metastasis, and prognosis (7–9). Data to support the independent prognostic value of genomics are currently sparse, however, and randomized controlled studies based on the presence of a specific variant have not been performed to demonstrate clinical utility for a variant-based treatment plan.

## Conclusions

Evaluation for genomic variants or fusions in DNA and/or RNA from thyroid nodule FNAs has been increasingly used to predict risks of malignancy in cytologically ITNs. However, only a few alterations (*BRAF^V600E^*, *BRAF^K601E^*, *NRAS^Q61R^*, *HRAS^Q61R^*, and *PAX8/PPARG*) have been reported in sufficient numbers from representative cohorts with histological confirmation to estimate meaningful predictive values. In this review, genomic alterations were present in a quarter of cytologically ITNs with *BRAF^V600E^* as the most common. However, *RAS* gene variants were the second most common, but data on specific variants in these and other genes were commonly not specified, making it impossible to determine accurate individual variant/fusion predictive values.

Furthermore, gene- or panel-level PPV, rather than individual variant or fusion-level PPV, may over- or underestimate the overall risk. To best estimate the true predictive and prognostic value of a specific genetic alteration, the cytological category, genomic and histological details for each individual variant/fusion from a cohort representative of those encountered in clinical practice need to be documented in a standard manner. The importance of accurate genomic variant designation will only continue to grow with the further advancements of precision medicine therapies.

## Supplementary Material

Supplemental data
