# Supplementary material for: Molecular Variants and Their Risks for Malignancy in Cytologically Indeterminate Thyroid Nodules
Source: Thyroid. 2019 Nov 14;29(11):1594–605. doi: 10.1089/thy.2019.0278 (PMC6864764; doi:10.1089/thy.2019.0278)
Supplement: Supplemental data [file Supp_Data.pdf]

Supplementary Data

SUPPLEMENTARY DATA S1. Search Criteria Used to Create List of Publications for Review

The search was conducted via https://www.ncbi.nlm.nih.gov/pubmed and using the ‘‘Advanced’’ tab a combination of terms to identify relevant publications:

all fields: (*PAX8* or translocation or variant or variants or mutation or mutations or molecular diagnostics or molecular testing or mutational panel or mutation analysis)
and

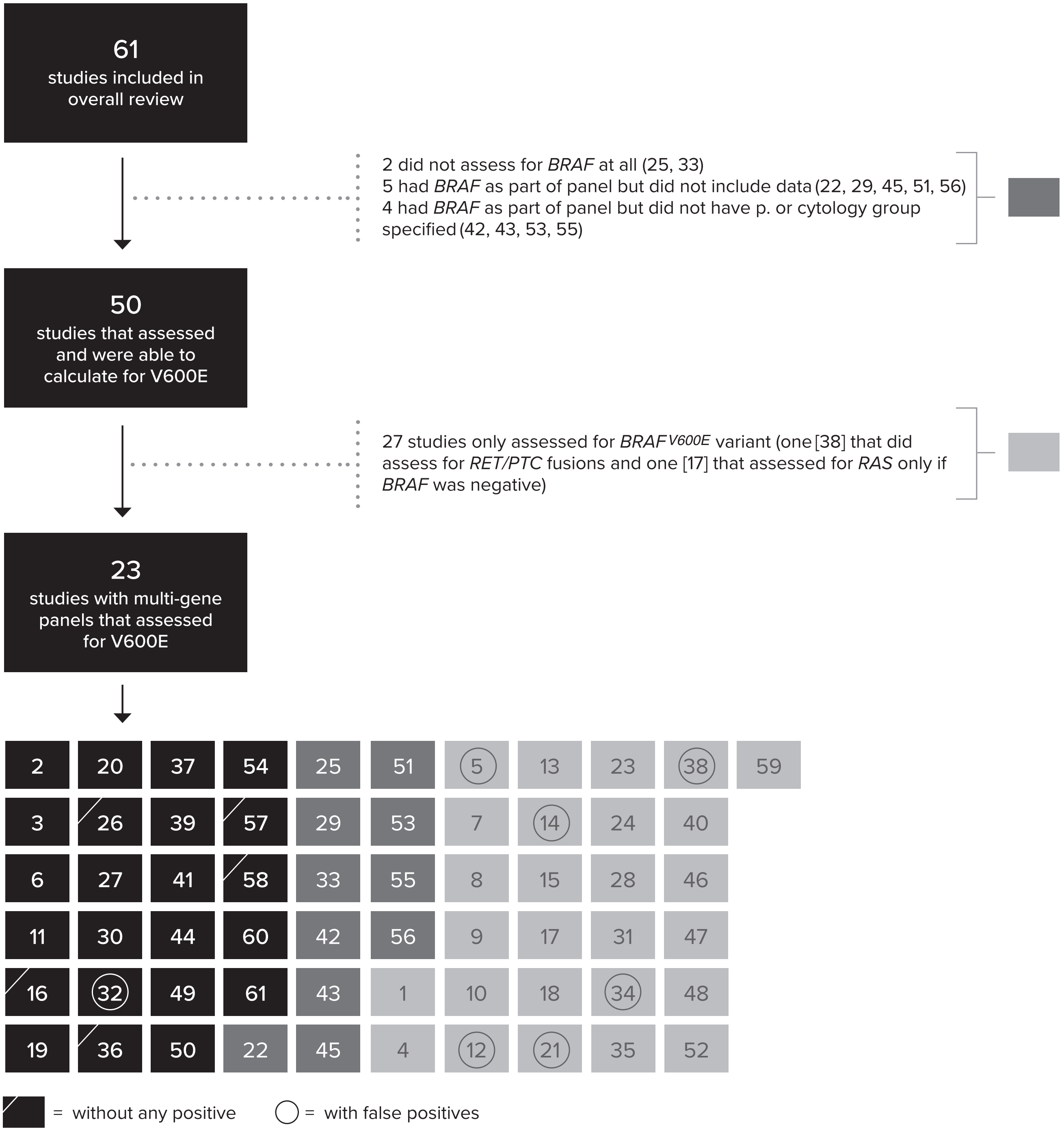

SUPPLEMENTARY FIG. S1. $BRAF^{V600E}$ data breakdown. As the most analyzed variant, this schematic shows how $BRAF^{V600E}$ was or was not evaluated across the 61 included studies. False positives were rare and were spread out across 7 different studies.

Supplementary Table S1. Included Studies

References	*Last name of lead author*	*Country*	*Year*	*Journal*	*Panel*	*Bethesda category*	*Sample type*	*Fusion*	*Multiples*
(S1)	Jo	Korea	2009	*Clin Endocrinol*	*BRAF*	III and IV	FNA	n/a	N
(S2)	Nikiforov	United States	2009	*JCEM*	7 Gene	III and IV	FNA	+	Y
(S3)	Moses	United States	2010	*World J Surg*	*BRAF*, *KRAS*, and *NRAS*	III and IV	FNA	+	N
(S4)	Adeniran	United States	2011	*Acta Cytol*	*BRAF*	III	FNA	n/a	N
(S5)	Kim	Korea	2011	*JCEM*	*BRAF*	III and IV	FNA	n/a	N
(S6)	Nikiforov	United States	2011	*JCEM*	7 Gene	III and IV	FNA	+	N
(S7)	Pelizzo	Europe	2011	*Clin Chem Lab Med*	BRAF	III and IV	FNA	n/a	N
(S8)	Yeo	Korea	2011	*Clin Endocrinol*	*BRAF*	III and IV	FNA	n/a	N
(S9)	Canadas-Garre	Europe	2012	*Ann Surg*	*BRAF*	III and IV	FNA	n/a	N
(S10)	Lee	Korea	2012	*JCEM*	*BRAF*	III and IV	FNA	n/a	N
(S11)	Mancini	Europe	2012	*J Mol Diagn*	7 Gene	III and IV	FNA	+	Y
(S12)	Jeong	Korea	2013	*AJR Am J Roentgenol*	*BRAF*	III	FNA	n/a	N
(S13)	Kloos	United States	2013	*JCEM*	*BRAF*	III and IV	FNA	n/a	N
(S14)	Koh	Korea	2013	*PLoS One*	*BRAF*	III and IV	FNA	n/a	N
(S15)	Agretti	Europe	2014	*J Endocrinol Invest*	*BRAF*	III	FNA	n/a	N
(S16)	Beaudenon-Huibregtse	United States	2014	*Thyroid*	7 Gene	III and IV	FNA	+	N
(S17)	Danilovic	Brazil	2014	*Eur J Endocrinol*	*BRAF*[a]	III and IV	Smear	n/a	N
(S18)	Johnson	Europe	2014	*Cytopathology*	*BRAF*	III and IV	Smear	n/a	N
(S19)	Liu	China	2014	*Exp Mol Pathol*	*BRAF*, *KRAS*, and *NRAS*	III and IV	FNA	–	N
(S20)	Nikiforov	United States	2014	*Cancer*	13 Gene	IV	FNA	+	Y
(S21)	Park	Korea	2014	*Cancer Cytopathol*	*BRAF*	III	FNA	n/a	N
(S22)	Radkay	United States	2014	*Cancer Cytopathol*	*HRAS*, *KRAS*, and *NRAS*[b]	III and IV	FNA	–	N
(S23)	Seo	Korea	2014	*Yonsei Med J*	*BRAF*	III and IV	FNA	n/a	N
(S24)	Seo	Korea	2014	*Endocrine*	*BRAF*	III and IV	FNA	n/a	N
(S25)	An	Korea	2015	*Clin Endocrinol*	*HRAS*, *KRAS*, and *NRAS*	III and IV	Smear	n/a	N
(S26)	Bongiovanni	Europe	2015	*Cytopathology*	7 Gene	IV	Smear	+	N
(S27)	Eszlinger	Europe	2015	*Thyroid*	7 Gene	III and IV	Smear	–	N
(S28)	Marino	Europe	2015	*Eur Thyroid J*	*BRAF*	III and IV	FNA	n/a	N
(S29)	Medici	United States	2015	*BMC Med*	*HRAS*, *KRAS*, and *NRAS*[b]	III and IV	FNA	+	N
(S30)	Nikiforov	United States	2015	*Thyroid*	14 Gene	III	FNA	+	N
(S31)	Park	Korea	2015	*J Mol Diagn*	*BRAF*	III and IV	FNA	n/a	N
(S32)	Stence	United States	2015	*Yale J Biol Med*	*BRAF*, *HRAS*, *KRAS*, and *NRAS*	III and IV	FNA	n/a	N
(S33)	Jang	Korea	2016	*Endocrine*	*NRAS*	III	Core	n/a	N
(S34)	Kim	Korea	2016	*Cancer Med*	*BRAF*	III	FNA	n/a	N
(S35)	Kowalska	Europe	2016	*Endokrynol Pol*	*BRAF*	III and IV	FNA	n/a	N
(S36)	Pagan	United States	2016	*BMC Bio-informatics*	524 Gene	III and IV	FNA	+	N
(S37)	Shrestha	United States	2016	*Thyroid*	14 Gene[c]	III and IV	FNA	+	Y
(S38)	Wei	China	2016	*PLoS One*	*BRAF*	III and IV	FNA	+	N
(S39)	Witt	United States	2016	*Del Med J*	14 Gene	III and IV	FNA	+	Y
(S40)	Beisa	Europe	2017	*Lange-nbecks Arch Surg*	*BRAF*	III and IV	FNA	n/a	N
(S41)	Censi	Europe	2017	*Front Endocrinol*	*BRAF*, *HRAS*, *KRAS*, *NRAS*, and *TERT*	III and IV	FNA	n/a	Y

(*continued*)

Supplementary Table S1. (Continued)

References	*Last name of lead author*	*Country*	*Year*	*Journal*	*Panel*	*Bethesda category*	*Sample type*	*Fusion*	*Multiples*
(S42)	Decaussin-Petrucci	Europe	2017	*Cytopathology*	*BRAF*, *HRAS*, *NRAS*, and *TERT*	III and IV	FNA	n/a	Y
(S43)	Eszlinger	Europe	2017	*Thyroid*	7 Gene	III and IV	Smear	+	N
(S44)	Giusti	Europe	2017	*J Zhejuang Univ Sci B*	*BRAF* and *NRAS*	III and IV	Smear	n/a	N
(S45)	Patel	United States	2017	*Surgery*	*HRAS*, *KRAS*, and *NRAS*[d]	III and IV	FNA	–	Y
(S46)	Rho	Korea	2017	*Ultrasound Q*	*BRAF*	III	FNA	n/a	N
(S47)	Rossi	Europe	2017	*Endocrine*	*BRAF*	III and IV	FNA	n/a	N
(S48)	Seo	Korea	2017	*Clin Otolaryngol*	*BRAF*	III	FNA	n/a	N
(S49)	Bellevicine	Europe	2018	*Cancer Cytopathol*	7 Gene	III	FNA	+	N
(S50)	Biron	Canada	2018	*J Oto-laryngol Head Neck Surg*	*BRAF*, *HRAS*, and *NRAS*	III and IV	FNA	n/a	N
(S51)	Guan	United States	2018	*Front Endocrinol*	*TSHR*[d]	III and IV	FNA	+	Y
(S52)	Kim	Korea	2018	*PLoS One*	BRAF	III and IV	FNA	n/a	N
(S53)	Livhits	United States	2018	*JCEM*	14 Gene	III and IV	FNA	+	N
(S54)	Macerola	Europe	2018	*J Endocrinol Invest*	*BRAF*, *HRAS*, *KRAS*, *NRAS*, and *TERT*	III and IV	FNA	n/a	N
(S55)	Maerki	United States	2018	*Diagn Cytopathol*	Some *BRAF*, *HRAS*, *KRAS*, and *NRAS* only and others 14 gene panel	III and IV	FNA	+	Y
(S56)	Mon	United States	2018	*Diagn Cytopathol*	*EZH1* and *TSHR*[e]	III and IV	FNA	n/a	N
(S57)	Partyka	United States	2018	*Diagn Cytopathol*	8 Gene	III and IV	FNA	–	N
(S58)	Rivas	United States	2018	*Endocr Pract*	14 Gene	III and IV	FNA	+	Y
(S59)	Rossi	Europe	2018	*Cytopathology*	*BRAF*	III and IV	FNA	n/a	N
(S60)	Taye	United States	2018	*Surgery*	14 Gene	III and IV	FNA	+	Y
(S61)	Valderrabano	United States	2018	*Head Neck*	14 Gene	III and IV	FNA	+	Y

Table contains list of studies reviewed that met inclusion criteria along with the respective publication and cohort specifics. For fusions: (n/a) represents no fusions were assessed, (–) indicates fusions were tested for but no positive nodules were noted, and (+) indicates at least one nodule was positive for a fusion. For multiples: (Y) for yes and (N) for no is used to designate if any nodules were found to have more than one variant. Supplementary Data S2 for detailed gene listing of each panel.

[a]*H*-, *K*-, and *N-RAS* were only evaluated if *BRAF* was negative and none was positive in indeterminate nodules.

[b]Although only the subset of genes listed were analyzed by the authors, full cohort was tested by full 7-gene panel.

[c]Some patients had 7-gene panel, and some had 14-gene panel, but authors found no significant difference in performance; therefore, data from both panels were combined.

[d]Although only the subset of genes listed were analyzed by the authors, full cohort was tested by full 14-gene panel.

[e]Although only the subset of genes listed were analyzed by the authors, full cohort was tested by full 12-gene panel.

FNA, fine-needle aspiration.

Supplementary Table S2. Excluded Studies

References	*Last name of lead author*	*Country*	*Year*	*Journal*	*Panel*
Cytology exclusions: cytology category could not be correlated with variant positives to ensure data were on AUS/FLUS and SFN/FN only					
(S62)	Girlando	Europe	2010	*Int J Surg Pathol*	*BRAF*
(S63)	Nam	South Korea	2010	*Thyroid*	*BRAF*
(S64)	Nikiforova	United States	2013	*JCEM*	12 Gene
(S65)	Eszlinger	Europe	2014	*Thyroid*	7 Gene
(S66)	Liu and Xing	United States	2014	*Endocr Relat Cancer*	*BRAF* and *TERT*
(S67)	Le Mercier	Europe	2015	*Histopathology*	50 Gene
(S68)	Paskas	Europe	2015	*Cancer Cytopathol*	BRAF
(S69)	Karunamurthy	United States	2016	*Endocr Relat Cancer*	14 Gene
(S70)	Wylie	United States	2016	*J Pathol Clin Res*	20 Gene
(S71)	Elsayed	Egypt	2018	*Int J Adv Res*	*NRAS*
(S72)	Gay	Europe	2018	*Med Sci Monit*	*BRAF*
(S73)	Jug	United States	2018	*Cancer Cytopathol*	7 Gene and 14 gene
(S74)	Steward	United States	2018	*JAMA Oncol*	112 Gene
Data extraction exclusions: unable to parse out and correlate positives, Bethesda category, and/or histological outcomes for data evaluation					
(S75)	Cantara	Europe	2010	*JCEM*	*BRAF*, *HRAS*, *KRAS*, and *NRAS*
(S76)	McCoy	United States	2012	*J Am Coll Surg*	7 Gene
(S77)	Rossi	Europe	2015	*Thyroid*	7 Gene
(S78)	Banizs	United States	2018	*Diagn Cytopathol*	8 Gene
Histology exclusions: histological outcomes were either not available or were not able to be correlated with mutation					
(S79)	Di Benedetto	Europe	2013	*Pathologica*	*BRAF*
(S80)	Hwang	South Korea	2015	*Biomed Res Int*	*BRAF*, *HRAS*, *KRAS*, and *NRAS*
(S81)	Monti	Europe	2015	*Int J Endocrinol*	*BRAF*
(S82)	Kwon	South Korea	2016	*Endocrinol Metab (Seoul)*	7 Gene
(S83)	Vuttariello	Europe	2017	*Oncotarget*	*BRAS*, *HRAS*, *KRAS*, *NRAS*, and *RET*
(S84)	Fuller	United States	2018	*Arch Pathol Lab Med*	50 Gene
(S85)	Marcadis	United States	2018 epub	*Surgery*	7 Gene and 14 gene
(S86)	Ohori	United States	2018	*Cancer Cytopathol*	112 Gene
Lab method exclusions: study stated concerns with lab method, which may have hindered accurate variant detection					
(S87)	Guerra	Europe	2011	*Endocr J*	PTC fusions
Language exclusions: study was written in language other than English					
(S88)	Dujardin	Europe	2010	*Ann Pathol*	*BRAF*
(S89)	Guerra	Europe	2013	*Recenti Prog Med*	*BRAF* and *RET/PTC* fusions only
(S90)	Wan	China	2014	*Zhonghua Er Bi Yan Hou Tou Jing Wai Ke Za Zhi*	*BRAF*

(*continued*)

Supplementary Table S2. (Continued)

References	*Last name of lead author*	*Country*	*Year*	*Journal*	*Panel*
Limited reporting exclusions: data were only reported for certain histological outcomes (e.g., PTC cases only)					
(S91)	Park	South Korea	2013	*Endocr Pathol*	*BRAF*, *HRAS*, *KRAS*, and *NRAS*
(S92)	Guo	China	2014	*Dis Markers*	*BRAF*, *HRAS*, *KRAS*, and *NRAS*
(S93)	Seo	South Korea	2015	*Ann Otol Rhinol Laryngol*	*BRAF*
(S94)	Nikiforova	United States	2018	*Cancer*	112 Gene
No positives exclusions: no variants were seen in AUS/FLUS or SFN/FN nodules					
(S95)	Marchetti	Europe	2009	*Thyroid*	*BRAF*
(S96)	Patel	Europe	2011	*Endocr Pathol*	*BRAF*
(S97)	Kang	South Korea	2012	*Cancer Cytopathol*	*BRAF*
(S98)	Brahma	Indonesia	2013	*Asian Pac J Cancer Prev*	*BRAF*
(S99)	Panebianco	Europe	2015	*BMC Cancer*	*BRAF*
(S100)	Shi	United States	2015	*Am J Clin Pathol*	*BRAF*
Overlap exclusions: high possibility of overlap between this cohort and other included studies					
(S101)	Zatelli	Europe	2009	*Eur J Endocrinol*	*BRAF*
(S102)	Ohori	United States	2010	*Cancer Cytopathol*	7 Gene
(S103)	Adeniran	United States	2011	*Thyroid*	*BRAF*
(S104)	Rossi	Europe	2012	*JCEM*	*BRAF*
(S105)	Carr	United states	2013	*Endocr Pathol*	*BRAF*
(S106)	Hyeon	South Korea	2014	*Cancer Cytopathol*	*BRAF*
(S107)	Beisa	Europe	2016	*Endokrynol Pol*	*BRAF*
(S108)	Borrelli	Europe	2016	*Cancer Cytopathol*	*BRAF*
(S109)	Valderrabano[a]	United States	2016	*Eur J Endocrinol*	13 Gene
(S110)	Valderrabano	United States	2017	*Endocr Relat Cancer*	14 Gene
Pediatric exclusions: samples were from pediatric populations					
(S111)	Buryk	United States	2013	*Int J Pediatr Otorhinolaryngol*	7 Gene
(S112)	Macerola	Europe	2017	*Int J Surg Pathol*	*BRAF*
(S113)	Monaco	United States	2012	*Cancer Cytopathol*	7 Gene
Review exclusions: review articles without novel cohort analysis included					
(S114)	Su	China	2016	*Onco Target Ther*	*BRAF*
(S115)	Klubo-Gwiezdzinska	United States	2018	*J Clin Endocrinol Metab*	Varied
Sample type exclusions: sample type was not presurgical FNA, slide scraping, or core biopsy					
(S116)	Kleiman	United States	2013	*Cancer*	*BRAF*

(*continued*)

Supplementary Table S2. (Continued)

References	Last name of lead author	Country	Year	Journal	Panel	
(S117)	Choi	South Korea	2015	*Thyroid*	*BRAF*	
(S118)	Gill	Canada	2015	*J Otolaryngol Head*	*BRAF*, *HRAS*, *KRAS*, and *NRAS*	
(S119)	De Napoli	Europe	2016	*Thyroid*	*BRAF* and *NRAS*	
(S120)	Crescenzi	Europe	2017	*Endocr Pathol*	*NRAS*	
Multiple exclusions: combination of above exclusion categories						
(S121)	Kim	Korea	2010	*JCEM*	*BRAF*	Lab Method and Overlap
(S122)	Mathur	United States	2010	*Surgery*	*BRAF*, *KRAS*, and *NRAS*	Cytology and Histology
(S123)	Ferraz and Pascke	Europe	2012	*Thyroid*	*PAX8/PPARG* and *RET/PTC* only	Lab Method and Overlap
(S124)	Moon	South Korea	2012	*AJR Am J Roentgenol*	*BRAF*	Cytology and Overlap
(S125)	Armstrong	United States	2013	*Thyroid*	*BRAF*, *HRAS*, *KRAS*, and *NRAS*	Histology and Overlap
(S126)	Gupta	United States	2013	*JCEM*	7 gene	Cytology, Histology and Overlap
(S127)	Ohori	United States	2013	*Cancer Cytopathol*	*BRAF*	Cytology, Limited Reporting and Overlap
(S128)	Krane	United States	2015	*Cancer Cytopathol*	7 Gene	Cytology and Histology
(S129)	Beisa	Europe	2018	*Exp Clin Endocrinol Diabetes*	*BRAF*	Limited Reporting and Overlap
(S130)	Valderrabano	United States	2018	*JAMA Otolaryngol Head Neck Surg*	7 gene and 14 gene	Histology and Overlap
(S131)	Zhang	China	2018	*Arch Med Res*	*BRAF*	Cytology and Limited Reporting

Table contains studies reviewed that did not meet inclusion criteria separated by their corresponding reason for exclusion. See Supplementary Data S2 for detailed gene listing of each panel.

[a]Although patients overlapped with included study (61) and was therefore excluded, variants were not fully specified in the later study so this publication was used to extract specific amino acid change wherever possible.

AUS, atypia of undetermined significance; FLUS, follicular lesion of undetermined significance; FN, follicular neoplasm; PTC, papillary thyroid cancer; SFN, suspicious for a follicular neoplasm, including Hürthle cell (oncocytic) type.

Supplementary Table S3. Suggested Standardized Chart for Future Data Collections

	Variant found	*Bethesda category*	*Total*	*Total operated*	*Histology results*
Variant example	*NRAS* p.Q61R	III	4	3	Follicular adenoma, NIFTP, PTC-follicular variant
Fusion example	*ETV6/NTRK3*	IV	1	1	Follicular thyroid cancer
Multiple example	*NRAS* p.Q61R and *TERT* C228T	V	1	1	PTC

Based on difficulties in extracting data from heterogeneous panel and publication data, it is suggested that future data on variants of indeterminate nodules specify the following data points for all positive nodules.

NIFTP, noninvasive follicular thyroid neoplasms with papillary-like nuclear features.

all fields: (fine-needle or aspiration or thyroid nodule or thyroid nodules or thyroid neoplasm or thyroid cancer or thyroid carcinoma)
and
all fields: (indeterminate or Bethesda III or Bethesda IV or pre-surgical or K601 or *RET/PTC* or AUS or FLUS)
NOT
publication type: (review)
Then click "Search"
Then under publication dates click "custom range" and enter (year/month/date) 2009/01/01 to 2018/12/31.

SUPPLEMENTARY DATA S2. Gene Panel Listing

Detailed list of genes analyzed in various panels of the publications reviewed is listed below. Other than the 7-gene panel, the fusions assessed varied from panel-to-panel and were not always outlined in totality within the study. Specific variants in each gene were also rarely listed.

7 Gene = *BRAF*, *NRAS*, *HRAS*, *KRAS*, *RET/PTC1*, *RET/PTC3*, *PAX8/PPARG*
8 Gene = 7-gene panel with *PIK3CA*
12 Gene = 8-gene panel with the addition of *AKT1*, *CTNNB1*, *GNAS*, *PIK3CA*, *PTEN*, *RET*, *TP53*, *TSHR*
13 Gene = 12-gene panel with the addition of *TERT*
14 Gene = 13-gene panel with the addition of *EIF1AX*
20 Gene = 14-gene panel with the addition of *ALK*, *APC*, *CDKN2A*, *IDH2*, *MET*, *SMAD4*
50 Gene = See reference (S84) for full listing
112 Gene = Neither reference (S74,S94) itemized the specific list of genes for this panel
524 Gene = See supplement of reference (S36) for full listing

Supplementary References

S1. Jo YS, Huang S, Kim YJ, Lee IS, Kim SS, Kim JR, Oh T, Moon Y, An S, Ro HK, Kim JM, Shong M 2009 Diagnostic value of pyrosequencing for the BRAF V600E mutation in ultrasound-guided fine-needle aspiration biopsy samples of thyroid incidentalomas. Clin Endocrinol (Oxf) **70:**139–144.
S2. Nikiforov YE, Steward DL, Robinson-Smith TM, Haugen BR, Klopper JP, Zhu Z, Fagin JA, Falciglia M, Weber K, Nikiforova MN 2009 Molecular testing for mutations in improving the fine-needle aspiration diagnosis of thyroid nodules. J Clin Endocrinol Metab **94:**2092–2098.
S3. Moses W, Weng J, Sansano I, Peng M, Khanafshar E, Ljung BM, Duh QY, Clark OH, Kebebew E 2010 Molecular testing for somatic mutations improves the accuracy of thyroid fine-needle aspiration biopsy. World J Surg **34:**2589–2594.
S4. Adeniran AJ, Hui P, Chhieng DC, Prasad ML, Schofield K, Theoharis C 2011 BRAF mutation testing of thyroid fine-needle aspiration specimens enhances the predictability of malignancy in thyroid follicular lesions of undetermined significance. Acta Cytol **55:**570–575.
S5. Kim SK, Hwang TS, Yoo YB, Han HS, Kim DL, Song KH, Lim SD, Kim WS, Paik NS 2011 Surgical results of thyroid nodules according to a management guideline based on the BRAF(V600E) mutation status. J Clin Endocrinol Metab **96:**658–664.
S6. Nikiforov YE, Ohori NP, Hodak SP, Carty SE, LeBeau SO, Ferris RL, Yip L, Seethala RR, Tublin ME, Stang MT, Coyne C, Johnson JT, Stewart AF, Nikiforova MN 2011 Impact of mutational testing on the diagnosis and management of patients with cytologically indeterminate thyroid nodules: a prospective analysis of 1056 FNA samples. J Clin Endocrinol Metab **96:**3390–3397.
S7. Pelizzo MR, Boschin IM, Barollo S, Pennelli G, Toniato A, Zambonin L, Vianello F, Piotto A, Ide EC, Pagetta C, Sorgato N, Torresan F, Girelli ME, Nacamulli D, Mantero F, Mian C 2011 BRAF analysis by fine needle aspiration biopsy of thyroid nodules improves preoperative identification of papillary thyroid carcinoma and represents a prognostic factor. A mono-institutional experience. Clin Chem Lab Med **49:**325–329.
S8. Yeo MK, Liang ZL, Oh T, Moon Y, An S, Kim MK, Kim KS, Shong M, Kim JM, Jo YS 2011 Pyrosequencing cut-off value identifying BRAFV600E mutation in fine needle aspiration samples of thyroid nodules. Clin Endocrinol (Oxf) **75:**555–560.
S9. Canadas-Garre M, Becerra-Massare P, Lopez de la Torre-Casares M, Villar-del Moral J, Cespedes-Mas S, Vilchez-Joya R, Muros-de Fuentes T, Garcia-Calvente C, Piedrola-Maroto G, Lopez-Nevot MA, Montes-Ramirez R, Llamas-Elvira JM 2012 Reduction of false-negative papillary thyroid carcinomas by the routine analysis of BRAF(T1799A) mutation on fine-needle aspiration biopsy specimens: a prospective study of 814 thyroid FNAB patients. Ann Surg **255:**986–992.
S10. Lee ST, Kim SW, Ki CS, Jang JH, Shin JH, Oh YL, Kim JW, Chung JH 2012 Clinical implication of highly sensitive detection of the BRAF V600E mutation in fine-needle aspirations of thyroid nodules: a comparative analysis of three molecular assays in 4585 consecutive

cases in a BRAF V600E mutation-prevalent area. J Clin Endocrinol Metab **97:**2299–2306.

S11. Mancini I, Pinzani P, Pupilli C, Petrone L, De Feo ML, Bencini L, Pazzagli M, Forti G, Orlando C 2012 A high-resolution melting protocol for rapid and accurate differential diagnosis of thyroid nodules. J Mol Diagn **14:**501–509.

S12. Jeong SH, Hong HS, Lee EH, Cha JG, Park JS, Kwak JJ 2013 Outcome of thyroid nodules characterized as atypia of undetermined significance or follicular lesion of undetermined significance and correlation with Ultrasound features and BRAF(V600E) mutation analysis. AJR Am J Roentgenol **201:**W854–W860.

S13. Kloos RT, Reynolds JD, Walsh PS, Wilde JI, Tom EY, Pagan M, Barbacioru C, Chudova DI, Wong M, Friedman L, LiVolsi VA, Rosai J, Lanman RB, Kennedy GC 2013 Does addition of BRAF V600E mutation testing modify sensitivity or specificity of the Afirma Gene Expression Classifier in cytologically indeterminate thyroid nodules? J Clin Endocrinol Metab **98:**E761–E768.

S14. Koh J, Choi JR, Han KH, Kim EK, Yoon JH, Moon HJ, Kwak JY 2013 Proper indication of BRAF(V600E) mutation testing in fine-needle aspirates of thyroid nodules. PLoS One **8:**e64505.

S15. Agretti P, Niccolai F, Rago T, De Marco G, Molinaro A, Scutari M, Di Cosmo C, Di Coscio G, Vitale M, Maccheroni M, Vitti P, Tonacchera M 2014 BRAF mutation analysis in thyroid nodules with indeterminate cytology: our experience on surgical management of patients with thyroid nodules from an area of borderline iodine deficiency. J Endocrinol Invest **37:**1009–1014.

S16. Beaudenon-Huibregtse S, Alexander EK, Guttler RB, Hershman JM, Babu V, Blevins TC, Moore P, Andruss B, Labourier E 2014 Centralized molecular testing for oncogenic gene mutations complements the local cytopathologic diagnosis of thyroid nodules. Thyroid **24:**1479–1487.

S17. Danilovic DL, Lima EU, Domingues RB, Brandao LG, Hoff AO, Marui S 2014 Pre-operative role of BRAF in the guidance of the surgical approach and prognosis of differentiated thyroid carcinoma. Eur J Endocrinol **170:**619–625.

S18. Johnson SJ, Hardy SA, Roberts C, Bourn D, Mallick U, Perros P 2014 Pilot of BRAF mutation analysis in indeterminate, suspicious and malignant thyroid FNA cytology. Cytopathology **25:**146–154.

S19. Liu S, Gao A, Zhang B, Zhang Z, Zhao Y, Chen P, Ji M, Hou P, Shi B 2014 Assessment of molecular testing in fine-needle aspiration biopsy samples: an experience in a Chinese population. Exp Mol Pathol **97:**292–297.

S20. Nikiforov YE, Carty SE, Chiosea SI, Coyne C, Duvvuri U, Ferris RL, Gooding WE, Hodak SP, LeBeau SO, Ohori NP, Seethala RR, Tublin ME, Yip L, Nikiforova MN 2014 Highly accurate diagnosis of cancer in thyroid nodules with follicular neoplasm/suspicious for a follicular neoplasm cytology by ThyroSeq v2 next-generation sequencing assay. Cancer **120:**3627–3634.

S21. Park HJ, Moon JH, Yom CK, Kim KH, Choi JY, Choi SI, Ahn SH, Jeong WJ, Lee WW, Park SY 2014 Thyroid ''atypia of undetermined significance'' with nuclear atypia has high rates of malignancy and BRAF mutation. Cancer Cytopathol **122:**512–520.

S22. Radkay LA, Chiosea SI, Seethala RR, Hodak SP, LeBeau SO, Yip L, McCoy KL, Carty SE, Schoedel KE, Nikiforova MN, Nikiforov YE, Ohori NP 2014 Thyroid nodules with KRAS mutations are different from nodules with NRAS and HRAS mutations with regard to cytopathologic and histopathologic outcome characteristics. Cancer Cytopathol **122:**873–882.

S23. Seo JY, Kim EK, Baek JH, Shin JH, Han KH, Kwak JY 2014 Can ultrasound be as a surrogate marker for diagnosing a papillary thyroid cancer? Comparison with BRAF mutation analysis. Yonsei Med J **55:**871–878.

S24. Seo JY, Kim EK, Kwak JY 2014 Additional BRAF mutation analysis may have additional diagnostic value in thyroid nodules with ''suspicious for malignant'' cytology alone even when the nodules do not show suspicious US features. Endocrine **47:**283–289.

S25. An JH, Song KH, Kim SK, Park KS, Yoo YB, Yang JH, Hwang TS, Kim DL 2015 RAS mutations in indeterminate thyroid nodules are predictive of the follicular variant of papillary thyroid carcinoma. Clin Endocrinol (Oxf) **82:**760–766.

S26. Bongiovanni M, Molinari F, Eszlinger M, Paschke R, Barizzi J, Merlo E, Giovanella L, Fasolini F, Cattaneo F, Ramelli F, Mazzucchelli L, Frattini M 2015 Laser capture microdissection is a valuable tool in the preoperative molecular screening of follicular lesions of the thyroid: an institutional experience. Cytopathology **26:**288–296.

S27. Eszlinger M, Piana S, Moll A, Bosenberg E, Bisagni A, Ciarrocchi A, Ragazzi M, Paschke R 2015 Molecular testing of thyroid fine-needle aspirations improves presurgical diagnosis and supports the histologic identification of minimally invasive follicular thyroid carcinomas. Thyroid **25:**401–409.

S28. Marino M, Monzani ML, Brigante G, Cioni K, Madeo B, Santi D, Maiorana A, Bettelli S, Moriondo V, Pignatti E, Bonacini L, Carani C, Rochira V, Simoni M 2015 High-resolution melting is a sensitive, cost-effective, time-saving technique for BRAF V600E detection in thyroid FNAB washing liquid: a prospective cohort study. Eur Thyroid J **4:**73–81.

S29. Medici M, Kwong N, Angell TE, Marqusee E, Kim MI, Frates MC, Benson CB, Cibas ES, Barletta JA, Krane JF, Ruan DT, Cho NL, Gawande AA, Moore FD, Jr., Alexander EK 2015 The variable phenotype and low-risk nature of RAS-positive thyroid nodules. BMC Med **13:**184.

S30. Nikiforov YE, Carty SE, Chiosea SI, Coyne C, Duvvuri U, Ferris RL, Gooding WE, LeBeau SO, Ohori NP, Seethala RR, Tublin ME, Yip L, Nikiforova MN 2015 Impact of the multi-gene ThyroSeq next-generation sequencing assay on cancer diagnosis in thyroid nodules with atypia of undetermined significance/follicular lesion of undetermined significance cytology. Thyroid **25:** 1217–1223.

S31. Park KS, Oh YL, Ki CS, Kim JW 2015 Evaluation of the Real-Q BRAF V600E Detection Assay in fine-needle aspiration samples of thyroid nodules. J Mol Diagn **17:**431–437.

S32. Stence AA, Gailey MP, Robinson RA, Jensen CS, Ma D 2015 Simultaneously detection of 50 mutations at 20 sites in the BRAF and RAS genes by multiplexed single-nucleotide primer extension assay using fine-needle aspirates of thyroid nodules. Yale J Biol Med **88:**351–358.

S33. Jang EK, Kim WG, Kim EY, Kwon H, Choi YM, Jeon MJ, Baek JH, Lee JH, Kim TY, Shong YK, Choi J, Song DE, Kim WB 2016 Usefulness of NRAS codon 61 mutation analysis and core needle biopsy for the diagnosis

of thyroid nodules previously diagnosed as atypia of undetermined significance. Endocrine **52:**305–312.
S34. Kim TH, Jeong DJ, Hahn SY, Shin JH, Oh YL, Ki CS, Kim JW, Jang JY, Cho YY, Chung JH, Kim SW 2016 Triage of patients with AUS/FLUS on thyroid cytopathology: effectiveness of the multimodal diagnostic techniques. Cancer Med **5:**769–777.
S35. Kowalska A, Kowalik A, Palyga I, Walczyk A, Gasior-Perczak D, Kopczynski J, Lizis-Kolus K, Szyska-Skrobot D, Hurej S, Radowicz-Chil A, Chodurska R, Wypiorkiewicz E, Chlopek M, Nowak E, Niemyska K, Gozdz S 2016 The usefulness of determining the presence of BRAF V600E mutation in fine-needle aspiration cytology in indeterminate cytological results. Endokrynol Pol **67:**41–47.
S36. Pagan M, Kloos RT, Lin CF, Travers KJ, Matsuzaki H, Tom EY, Kim SY, Wong MG, Stewart AC, Huang J, Walsh PS, Monroe RJ, Kennedy GC 2016 The diagnostic application of RNA sequencing in patients with thyroid cancer: an analysis of 851 variants and 133 fusions in 524 genes. BMC Bioinformatics **17(Suppl. 1):**6.
S37. Shrestha RT, Evasovich MR, Amin K, Radulescu A, Sanghvi TS, Nelson AC, Shahi M, Burmeister LA 2016 Correlation between histological diagnosis and mutational panel testing of thyroid nodules: a two-year institutional experience. Thyroid **26:**1068–1076.
S38. Wei Y, Zhou X, Liu S, Wang H, Liu L, Liu R, Kang J, Hong K, Wang D, Yuan G 2016 novel and practical scoring systems for the diagnosis of thyroid nodules. PLoS One **11:**e0163039.
S39. Witt RL 2016 Targeted next generation sequencing with ThyroSeq v2.1 for indeterminate thyroid nodules in clinical practice. Del Med J **88:**366–372.
S40. Beisa A, Kvietkauskas M, Beisa V, Stoskus M, Ostaneviciute E, Jasiunas E, Griskevicius L, Strupas K 2017 The utility of the Bethesda category and its association with BRAF mutation in the prediction of papillary thyroid cancer stage. Langenbecks Arch Surg **402:**227–234.
S41. Censi S, Cavedon E, Bertazza L, Galuppini F, Watutantrige-Fernando S, De Lazzari P, Nacamulli D, Pennelli G, Fassina A, Iacobone M, Casal Ide E, Vianello F, Barollo S, Mian C 2017 Frequency and significance of Ras, Tert promoter, and Braf mutations in cytologically indeterminate thyroid nodules: a monocentric case series at a tertiary-level endocrinology unit. Front Endocrinol (Lausanne) **8:**273.
S42. Decaussin-Petrucci M, Descotes F, Depaepe L, Lapras V, Denier ML, Borson-Chazot F, Lifante JC, Lopez J 2017 Molecular testing of BRAF, RAS and TERT on thyroid FNAs with indeterminate cytology improves diagnostic accuracy. Cytopathology **28:**482–487.
S43. Eszlinger M, Bohme K, Ullmann M, Gorke F, Siebolts U, Neumann A, Franzius C, Adam S, Molwitz T, Landvogt C, Amro B, Hach A, Feldmann B, Graf D, Wefer A, Niemann R, Bullmann C, Klaushenke G, Santen R, Tonshoff G, Ivancevic V, Kogler A, Bell E, Lorenz B, Kluge G, Hartenstein C, Ruschenburg I, Paschke R 2017 Evaluation of a two-year routine application of molecular testing of thyroid fine-needle aspirations using a seven-gene panel in a primary referral setting in Germany. Thyroid **27:**402–411.
S44. Giusti M, Massa B, Balestra M, Calamaro P, Gay S, Schiaffino S, Turtulici G, Zupo S, Monti E, Ansaldo G 2017 Retrospective cytological evaluation of indeterminate thyroid nodules according to the British Thyroid Association 2014 classification and comparison of clinical evaluation and outcomes. J Zhejiang Univ Sci B **18:**555–566.
S45. Patel SG, Carty SE, McCoy KL, Ohori NP, LeBeau SO, Seethala RR, Nikiforova MN, Nikiforov YE, Yip L 2017 Preoperative detection of RAS mutation may guide extent of thyroidectomy. Surgery **161:**168–175.
S46. Rho M, Kim EK, Moon HJ, Yoon JH, Park VY, Han K, Kwak JY 2017 Clinical parameter for deciding the BRAFV600E mutation test in atypia of undetermined significance/follicular lesion of undetermined significance thyroid nodules: US features according to TIRADS. Ultrasound Q **33:**284–288.
S47. Rossi M, Lupo S, Rossi R, Franceschetti P, Trasforini G, Bruni S, Tagliati F, Buratto M, Lanza G, Damiani L, Degli Uberti E, Zatelli MC 2017 Proposal for a novel management of indeterminate thyroid nodules on the basis of cytopathological subclasses. Endocrine **57:**98–107.
S48. Seo JW, Jang AL, Suh SH, Park HS, Kang MK, Hong JC 2017 Atypia of undetermined significance on thyroid fine needle aspiration—risk factors for malignancy. Clin Otolaryngol **42:**234–238.
S49. Bellevicine C, Sgariglia R, Migliatico I, Vigliar E, D'Anna M, Nacchio MA, Serra N, Malapelle U, Bongiovanni M, Troncone G 2018 Different qualifiers of AUS/FLUS thyroid FNA have distinct BRAF, RAS, RET/PTC, and PAX8/PPARg alterations. Cancer Cytopathol **126:**317–325.
S50. Biron VL, Matkin A, Kostiuk M, Williams J, Cote DW, Harris J, Seikaly H, O'Connell DA 2018 Analytic and clinical validity of thyroid nodule mutational profiling using droplet digital polymerase chain reaction. J Otolaryngol Head Neck Surg **47:**60.
S51. Guan H, Matonis D, Toraldo G, Lee SL 2018 Clinical significance of thyroid-stimulating hormone receptor gene mutations and/or sodium-iodine symporter gene overexpression in indeterminate thyroid fine needle biopsies. Front Endocrinol (Lausanne) **9:**566.
S52. Kim DS, Kim DW, Heo YJ, Baek JW, Lee YJ, Choo HJ, Park YM, Park HK, Ha TK, Kim DH, Jung SJ, Park JS, Ahn KJ, Baek HJ, Kang T 2018 Utility of including BRAF mutation analysis with ultrasonographic and cytological diagnoses in ultrasonography-guided fine-needle aspiration of thyroid nodules. PLoS One **13:**e0202687.
S53. Livhits MJ, Kuo EJ, Leung AM, Rao J, Levin M, Douek ML, Beckett KR, Zanocco KA, Cheung DS, Gofnung YA, Smooke-Praw S, Yeh MW 2018 Gene expression classifier vs targeted next-generation sequencing in the management of indeterminate thyroid nodules. J Clin Endocrinol Metab **103:**2261–2268.
S54. Macerola E, Rago T, Proietti A, Basolo F, Vitti P 2019 The mutational analysis in the diagnostic work-up of thyroid nodules: the real impact in a center with large experience in thyroid cytopathology. J Endocrinol Invest **42:**157–166.
S55. Maerki J, Klein M, Chau K, Gimenez C, Fishbein J, Khutti S, Das K 2019 Determining the molecular test for indeterminate thyroid nodules best suited for our practice: a quality assurance study. Diagn Cytopathol **47:**259–267.
S56. Mon SY, Riedlinger G, Abbott CE, Seethala R, Ohori NP, Nikiforova MN, Nikiforov YE, Hodak SP 2018 Cancer risk and clinicopathological characteristics of thyroid nodules harboring thyroid-stimulating hormone receptor gene mutations. Diagn Cytopathol **46:**369–377.

S57. Partyka KL, Randolph ML, Lawrence KA, Cramer H, Wu HH 2018 Utilization of direct smears of thyroid fine-needle aspirates for ancillary molecular testing: a comparison of two proprietary testing platforms. Diagn Cytopathol **46:**320–325.
S58. Rivas AM, Nassar A, Zhang J, Casler JD, Chindris AM, Smallridge R, Bernet V 2018 ThyroSeq((R))V2.0 molecular testing: a cost-effective approach for the evaluation of indeterminate thyroid nodules. Endocr Pract **24:**780–788.
S59. Rossi ED, Martini M, Capodimonti S, Cenci T, Bilotta M, Pierconti F, Pontecorvi A, Lombardi CP, Fadda G, Larocca LM 2018 Morphology combined with ancillary techniques: an algorithm approach for thyroid nodules. Cytopathology **29:**418–427.
S60. Taye A, Gurciullo D, Miles BA, Gupta A, Owen RP, Inabnet WB, 3rd, Beyda JN, Marti JL 2018 Clinical performance of a next-generation sequencing assay (ThyroSeq v2) in the evaluation of indeterminate thyroid nodules. Surgery **163:**97–103.
S61. Valderrabano P, Khazai L, Thompson ZJ, Leon ME, Otto KJ, Hallanger-Johnson JE, Wadsworth JT, Chung CH, Centeno BA, McIver B 2018 Impact of oncogene panel results on surgical management of cytologically indeterminate thyroid nodules. Head Neck **40:**1812–1823.
S62. Girlando S, Cuorvo LV, Bonzanini M, Morelli L, Amadori P, Dalla Palma P, Barbareschi M 2010 High prevalence of B-RAF mutation in papillary carcinoma of the thyroid in north-east Italy. Int J Surg Pathol **18:**173–176.
S63. Nam SY, Han BK, Ko EY, Kang SS, Hahn SY, Hwang JY, Nam MY, Kim JW, Chung JH, Oh YL, Shin JH 2010 BRAF V600E mutation analysis of thyroid nodules needle aspirates in relation to their ultrasongraphic classification: a potential guide for selection of samples for molecular analysis. Thyroid **20:**273–279.
S64. Nikiforova MN, Wald AI, Roy S, Durso MB, Nikiforov YE 2013 Targeted next-generation sequencing panel (ThyroSeq) for detection of mutations in thyroid cancer. J Clin Endocrinol Metab **98:**E1852–E1860.
S65. Eszlinger M, Krogdahl A, Munz S, Rehfeld C, Precht Jensen EM, Ferraz C, Bosenberg E, Drieschner N, Scholz M, Hegedus L, Paschke R 2014 Impact of molecular screening for point mutations and rearrangements in routine air-dried fine-needle aspiration samples of thyroid nodules. Thyroid **24:**305–313.
S66. Liu R, Xing M 2014 Diagnostic and prognostic TERT promoter mutations in thyroid fine-needle aspiration biopsy. Endocr Relat Cancer **21:**825–830.
S67. Le Mercier M, D'Haene N, De Neve N, Blanchard O, Degand C, Rorive S, Salmon I 2015 Next-generation sequencing improves the diagnosis of thyroid FNA specimens with indeterminate cytology. Histopathology **66:**215–224.
S68. Paskas S, Jankovic J, Zivaljevic V, Tatic S, Bozic V, Nikolic A, Radojkovic D, Savin S, Cvejic D 2015 Malignant risk stratification of thyroid FNA specimens with indeterminate cytology based on molecular testing. Cancer Cytopathol **123:**471–479.
S69. Karunamurthy A, Panebianco F, S JH, Vorhauer J, Nikiforova MN, Chiosea S, Nikiforov YE 2016 Prevalence and phenotypic correlations of EIF1AX mutations in thyroid nodules. Endocr Relat Cancer **23:**295–301.
S70. Wylie D, Beaudenon-Huibregtse S, Haynes BC, Giordano TJ, Labourier E 2016 Molecular classification of thyroid lesions by combined testing for miRNA gene expression and somatic gene alterations. J Pathol Clin Res **2:**93–103.
S71. Elsayed YA, El-Latif AMA, Zaghloul MHE, Lofty SE, Mahmoud MM 2018 Diagnostic and prognostic utility of NRAS mutation gene testing in cytologically indeterminate thyroid nodules. Int J Adv Res **6:**744–753.
S72. Gay S, Schiaffino S, Santamorena G, Massa B, Ansaldo G, Turtulici G, Giusti M, At The Policlinico San Martino Genoa TT 2018 Role of strain elastography and shear-wave elastography in a multiparametric clinical approach to indeterminate cytology thyroid nodules. Med Sci Monit **24:**6273–6279.
S73. Jug RC, Datto MB, Jiang XS 2018 Molecular testing for indeterminate thyroid nodules: performance of the Afirma gene expression classifier and ThyroSeq panel. Cancer Cytopathol **126:**471–480.
S74. Steward DL, Carty SE, Sippel RS, Yang SP, Sosa JA, Sipos JA, Figge JJ, Mandel S, Haugen BR, Burman KD, Baloch ZW, Lloyd RV, Seethala RR, Gooding WE, Chiosea SI, Gomes-Lima C, Ferris RL, Folek JM, Khawaja RA, Kundra P, Loh KS, Marshall CB, Mayson S, McCoy KL, Nga ME, Ngiam KY, Nikiforova MN, Poehls JL, Ringel MD, Yang H, Yip L, Nikiforov YE 2018 Performance of a multigene genomic classifier in thyroid nodules with indeterminate cytology: a prospective blinded multicenter study. JAMA Oncol **5:**204–212.
S75. Cantara S, Capezzone M, Marchisotta S, Capuano S, Busonero G, Toti P, Di Santo A, Caruso G, Carli AF, Brilli L, Montanaro A, Pacini F 2010 Impact of proto-oncogene mutation detection in cytological specimens from thyroid nodules improves the diagnostic accuracy of cytology. J Clin Endocrinol Metab **95:**1365–1369.
S76. McCoy KL, Carty SE, Armstrong MJ, Seethala RR, Ohori NP, Kabaker AS, Stang MT, Hodak SP, Nikiforov YE, Yip L 2012 Intraoperative pathologic examination in the era of molecular testing for differentiated thyroid cancer. J Am Coll Surg **215:**546–554.
S77. Rossi M, Buratto M, Tagliati F, Rossi R, Lupo S, Trasforini G, Lanza G, Franceschetti P, Bruni S, Degli Uberti E, Zatelli MC 2015 Relevance of BRAF(V600E) mutation testing versus RAS point mutations and RET/PTC rearrangements evaluation in the diagnosis of thyroid cancer. Thyroid **25:**221–228.
S78. Banizs AB, Silverman JF 2019 The utility of combined mutation analysis and microRNA classification in reclassifying cancer risk of cytologically indeterminate thyroid nodules. Diagn Cytopathol **47:**268–274.
S79. Di Benedetto G, Fabozzi A, Rinaldi C 2013 Clinical management of thyroid nodules with indeterminate cytology: our institutional experience using SIAPEC cytological criteria and V600-BRAF test. Pathologica **105:**1–4.
S80. Hwang TS, Kim WY, Han HS, Lim SD, Kim WS, Yoo YB, Park KS, Oh SY, Kim SK, Yang JH 2015 Preoperative RAS mutational analysis is of great value in predicting follicular variant of papillary thyroid carcinoma. Biomed Res Int **2015:**697068.
S81. Monti E, Bovero M, Mortara L, Pera G, Zupo S, Gugiatti E, Dono M, Massa B, Ansaldo GL, Massimo G 2015 BRAF Mutations in an Italian Regional Population: implications for the therapy of thyroid cancer. Int J Endocrinol **2015:**138734.

S82. Kwon H, Kim WG, Eszlinger M, Paschke R, Song DE, Kim M, Park S, Jeon MJ, Kim TY, Shong YK, Kim WB 2016 Molecular diagnosis using residual liquid-based cytology materials for patients with nondiagnostic or indeterminate thyroid nodules. Endocrinol Metab (Seoul) **31:**586–591.

S83. Vuttariello E, Borra M, Mauriello E, Calise C, D'Andrea B, Capiluongo A, Fulciniti F, Cipolletta A, Monaco M, Pezzullo L, Chiappetta G 2017 Multiplex PCR approach to simultaneously identify several mutations in fine needle cytology thyroid samples. Oncotarget **8:**49351–49358.

S84. Fuller MY, Mody D, Hull A, Pepper K, Hendrickson H, Olsen R 2018 Next-generation sequencing identifies gene mutations that are predictive of malignancy in residual needle rinses collected from fine-needle aspirations of thyroid nodules. Arch Pathol Lab Med **142:**178–183.

S85. Marcadis AR, Valderrabano P, Ho AS, Tepe J, Swartzwelder CE, Byrd S, Sacks WL, Untch BR, Shaha AR, Xu B, Lin O, Ghossein RA, Wong RJ, Marti JL, Morris LGT 2019 Interinstitutional variation in predictive value of the ThyroSeq v2 genomic classifier for cytologically indeterminate thyroid nodules. Surgery **165:**17–24.

S86. Ohori NP, Landau MS, Carty SE, Yip L, LeBeau SO, Manroa P, Seethala RR, Schoedel KE, Nikiforova MN, Nikiforov YE 2019 Benign call rate and molecular test result distribution of ThyroSeq v3. Cancer Cytopathol **127:**161–168.

S87. Guerra A, Sapio MR, Marotta V, Campanile E, Moretti MI, Deandrea M, Motta M, Limone PP, Fenzi G, Rossi G, Vitale M 2011 Prevalence of RET/PTC rearrangement in benign and malignant thyroid nodules and its clinical application. Endocr J **58:**31–38.

S88. Dujardin F, Pages JC, Collin C, de Calan L, Lecomte P, Guyetant S 2010 [BRAF V600E mutation in papillary thyroid carcinoma: prevalence and detection in fine needle aspiration specimens]. Ann Pathol **30:**252–262.

S89. Guerra A, Carrano M, Angrisani E, Vitale M 2013 [Diagnostic value of BRAFV600E and RET/PTC oncogenes in thyroid nodule aspirates]. Recenti Prog Med **104:**415–419.

S90. Wan H, Zhang B, Wang Y, Xiao T, Guo H, Liu W, Yan D, Xu Z, Tang P 2014 [Clinical role of BRAF V600E mutation testing in thyroid nodules]. Zhonghua Er Bi Yan Hou Tou Jing Wai Ke Za Zhi **49:**468–472.

S91. Park JY, Kim WY, Hwang TS, Lee SS, Kim H, Han HS, Lim SD, Kim WS, Yoo YB, Park KS 2013 BRAF and RAS mutations in follicular variants of papillary thyroid carcinoma. Endocr Pathol **24:**69–76.

S92. Guo HQ, Zhao H, Zhang ZH, Zhu YL, Xiao T, Pan QJ 2014 Impact of molecular testing in the diagnosis of thyroid fine needle aspiration cytology: data from mainland China. Dis Markers **2014:**912182.

S93. Seo JY, Choi JR, Moon HJ, Kim EK, Han KH, Kim H, Kwak JY 2015 Clinical implication of highly sensitive detection of the BRAFV600E mutation in fine-needle aspirations according to the thyroid Bethesda system in patients with conventional papillary thyroid carcinoma. Ann Otol Rhinol Laryngol **124:**392–399.

S94. Nikiforova MN, Mercurio S, Wald AI, Barbi de Moura M, Callenberg K, Santana-Santos L, Gooding WE, Yip L, Ferris RL, Nikiforov YE 2018 Analytical performance of the ThyroSeq v3 genomic classifier for cancer diagnosis in thyroid nodules. Cancer **124:**1682–1690.

S95. Marchetti I, Lessi F, Mazzanti CM, Bertacca G, Elisei R, Coscio GD, Pinchera A, Bevilacqua G 2009 A morpho-molecular diagnosis of papillary thyroid carcinoma: BRAF V600E detection as an important tool in preoperative evaluation of fine-needle aspirates. Thyroid **19:**837–842.

S96. Patel A, Klubo-Gwiezdzinska J, Hoperia V, Larin A, Jensen K, Bauer A, Vasko V 2011 BRAF(V600E) mutation analysis from May-Grunwald Giemsa-stained cytological samples as an adjunct in identification of high-risk papillary thyroid carcinoma. Endocr Pathol **22:**195–199.

S97. Kang G, Cho EY, Shin JH, Chung JH, Kim JW, Oh YL 2012 Role of BRAFV600E mutation analysis and second cytologic review of fine-needle aspiration for evaluating thyroid nodule. Cancer Cytopathol **120:**44–51.

S98. Brahma B, Yulian ED, Ramli M, Setianingsih I, Gautama W, Brahma P, Sastroasmoro S, Harimurti K 2013 Surgical perspective of T1799A BRAF mutation diagnostic value in papillary thyroid carcinoma. Asian Pac J Cancer Prev **14:**31–37.

S99. Panebianco F, Mazzanti C, Tomei S, Aretini P, Franceschi S, Lessi F, Di Coscio G, Bevilacqua G, Marchetti I 2015 The combination of four molecular markers improves thyroid cancer cytologic diagnosis and patient management. BMC Cancer **15:**918.

S100. Shi Q, Ibrahim A, Herbert K, Carvin M, Randolph M, Post KM, Curless K, Chen S, Cramer HM, Cheng L, Wu HH 2015 Detection of BRAF mutations on direct smears of thyroid fine-needle aspirates through cell transfer technique. Am J Clin Pathol **143:**500–504.

S101. Zatelli MC, Trasforini G, Leoni S, Frigato G, Buratto M, Tagliati F, Rossi R, Cavazzini L, Roti E, degli Uberti EC 2009 BRAF V600E mutation analysis increases diagnostic accuracy for papillary thyroid carcinoma in fine-needle aspiration biopsies. Eur J Endocrinol **161:**467–473.

S102. Ohori NP, Nikiforova MN, Schoedel KE, LeBeau SO, Hodak SP, Seethala RR, Carty SE, Ogilvie JB, Yip L, Nikiforov YE 2010 Contribution of molecular testing to thyroid fine-needle aspiration cytology of "follicular lesion of undetermined significance/atypia of undetermined significance". Cancer Cytopathol **118:**17–23.

S103. Adeniran AJ, Theoharis C, Hui P, Prasad ML, Hammers L, Carling T, Udelsman R, Chhieng DC 2011 Reflex BRAF testing in thyroid fine-needle aspiration biopsy with equivocal and positive interpretation: a prospective study. Thyroid **21:**717–723.

S104. Rossi M, Buratto M, Bruni S, Filieri C, Tagliati F, Trasforini G, Rossi R, Beccati MD, Degli Uberti EC, Zatelli MC 2012 Role of ultrasonographic/clinical profile, cytology, and BRAF V600E mutation evaluation in thyroid nodule screening for malignancy: a prospective study. J Clin Endocrinol Metab **97:**2354–2361.

S105. Carr R, Ustun B, Chhieng D, Schofield K, Theoharis C, Hammers L, Adeniran AJ 2013 Radiologic and clinical predictors of malignancy in the follicular lesion of undetermined significance of the thyroid. Endocr Pathol **24:**62–68.

S106. Hyeon J, Ahn S, Shin JH, Oh YL 2014 The prediction of malignant risk in the category "atypia of undetermined significance/follicular lesion of undetermined significance" of the Bethesda System for Reporting Thyroid Cytopathology using subcategorization and BRAF mutation results. Cancer Cytopathol **122:**368–376.

S107. Beisa A, Beisa V, Stoskus M, Ostaneviciute E, Griskevicius L, Strupas K 2016 The value of the repeated ex-

amination of BRAF V600E mutation status in diagnostics of papillary thyroid cancer. Endokrynol Pol **67:**35–40.

S108. Borrelli N, Ugolini C, Giannini R, Antonelli A, Giordano M, Sensi E, Torregrossa L, Fallahi P, Miccoli P, Basolo F 2016 Role of gene expression profiling in defining indeterminate thyroid nodules in addition to BRAF analysis. Cancer Cytopathol **124:**340–349.

S109. Valderrabano P, Leon ME, Centeno BA, Otto KJ, Khazai L, McCaffrey JC, Russell JS, McIver B 2016 Institutional prevalence of malignancy of indeterminate thyroid cytology is necessary but insufficient to accurately interpret molecular marker tests. Eur J Endocrinol **174:**621–629.

S110. Valderrabano P, Khazai L, Leon ME, Thompson ZJ, Ma Z, Chung CH, Hallanger-Johnson JE, Otto KJ, Rogers KD, Centeno BA, McIver B 2017 Evaluation of ThyroSeq v2 performance in thyroid nodules with indeterminate cytology. Endocr Relat Cancer **24:**127–136.

S111. Buryk MA, Monaco SE, Witchel SF, Mehta DK, Gurtunca N, Nikiforov YE, Simons JP 2013 Preoperative cytology with molecular analysis to help guide surgery for pediatric thyroid nodules. Int J Pediatr Otorhinolaryngol **77:**1697–1700.

S112. Macerola E, Torregrossa L, Ugolini C, Bakkar S, Vitti P, Fadda G, Basolo F 2017 BRAF(K601E) mutation in a follicular thyroid adenoma: a case report. Int J Surg Pathol **25:**348–351.

S113. Monaco SE, Pantanowitz L, Khalbuss WE, Benkovich VA, Ozolek J, Nikiforova MN, Simons JP, Nikiforov YE 2012 Cytomorphological and molecular genetic findings in pediatric thyroid fine-needle aspiration. Cancer Cytopathol **120:**342–350.

S114. Su X, Jiang X, Xu X, Wang W, Teng X, Shao A, Teng L 2016 Diagnostic value of BRAF (V600E)-mutation analysis in fine-needle aspiration of thyroid nodules: a meta-analysis. Onco Targets Ther **9:**2495–2509.

S115. Klubo-Gwiezdzinska J, Wartofsky L 2018 The role of molecular diagnostics in the management of indeterminate thyroid nodules. J Clin Endocrinol Metab **103:**3507–3510.

S116. Kleiman DA, Sporn MJ, Beninato T, Crowley MJ, Nguyen A, Uccelli A, Scognamiglio T, Zarnegar R, Fahey TJ, 3rd 2013 Preoperative BRAF(V600E) mutation screening is unlikely to alter initial surgical treatment of patients with indeterminate thyroid nodules: a prospective case series of 960 patients. Cancer **119:**1495–1502.

S117. Choi SH, Baek JH, Lee JH, Choi YJ, Song DE, Chung KW, Kim TY, Shong YK 2015 Evaluation of the clinical usefulness of BRAFV600E mutation analysis of core-needle biopsy specimens in thyroid nodules with previous atypia of undetermined significance or follicular lesions of undetermined significance results. Thyroid **25:**897–903.

S118. Gill MS, Nayan S, Kocovski L, Cutz JC, Archibald SD, Jackson BS, Young JE, Gupta MK 2015 Local molecular analysis of indeterminate thyroid nodules. J Otolaryngol Head Neck Surg **44:**52.

S119. De Napoli L, Bakkar S, Ambrosini CE, Materazzi G, Proietti A, Macerola E, Basolo F, Miccoli P 2016 Indeterminate single thyroid nodule: synergistic impact of mutational markers and sonographic features in triaging patients to appropriate surgery. Thyroid **26:**390–394.

S120. Crescenzi A, Fulciniti F, Bongiovanni M, Giovanella L, Trimboli P 2017 Detecting N-RAS Q61R mutated thyroid neoplasias by immunohistochemistry. Endocr Pathol **28:**71–74.

S121. Kim SW, Lee JI, Kim JW, Ki CS, Oh YL, Choi YL, Shin JH, Kim HK, Jang HW, Chung JH 2010 BRAFV600E mutation analysis in fine-needle aspiration cytology specimens for evaluation of thyroid nodule: a large series in a BRAFV600E-prevalent population. J Clin Endocrinol Metab **95:**3693–3700.

S122. Mathur A, Weng J, Moses W, Steinberg SM, Rahbari R, Kitano M, Khanafshar E, Ljung BM, Duh QY, Clark OH, Kebebew E 2010 A prospective study evaluating the accuracy of using combined clinical factors and candidate diagnostic markers to refine the accuracy of thyroid fine needle aspiration biopsy. Surgery **148:**1170–1176; discussion 1176–1177.

S123. Ferraz C, Rehfeld C, Krogdahl A, Precht Jensen EM, Bosenberg E, Narz F, Hegedus L, Paschke R, Eszlinger M 2012 Detection of PAX8/PPARG and RET/PTC rearrangements is feasible in routine air-dried fine needle aspiration smears. Thyroid **22:**1025–1030.

S124. Moon WJ, Choi N, Choi JW, Kim SK, Hwang TS 2012 BRAF mutation analysis and sonography as adjuncts to fine-needle aspiration cytology of papillary thyroid carcinoma: their relationships and roles. AJR Am J Roentgenol **198:**668–674.

S125. Armstrong MJ, Yang H, Yip L, Ohori NP, McCoy KL, Stang MT, Hodak SP, Nikiforova MN, Carty SE, Nikiforov YE 2014 PAX8/PPARgamma rearrangement in thyroid nodules predicts follicular-pattern carcinomas, in particular the encapsulated follicular variant of papillary carcinoma. Thyroid **24:**1369–1374.

S126. Gupta N, Dasyam AK, Carty SE, Nikiforova MN, Ohori NP, Armstrong M, Yip L, LeBeau SO, McCoy KL, Coyne C, Stang MT, Johnson J, Ferris RL, Seethala R, Nikiforov YE, Hodak SP 2013 RAS mutations in thyroid FNA specimens are highly predictive of predominantly low-risk follicular-pattern cancers. J Clin Endocrinol Metab **98:**E914–E922.

S127. Ohori NP, Singhal R, Nikiforova MN, Yip L, Schoedel KE, Coyne C, McCoy KL, LeBeau SO, Hodak SP, Carty SE, Nikiforov YE 2013 BRAF mutation detection in indeterminate thyroid cytology specimens: underlying cytologic, molecular, and pathologic characteristics of papillary thyroid carcinoma. Cancer Cytopathol **121:**197–205.

S128. Krane JF, Cibas ES, Alexander EK, Paschke R, Eszlinger M 2015 Molecular analysis of residual ThinPrep material from thyroid FNAs increases diagnostic sensitivity. Cancer Cytopathol **123:**356–361.

S129. Beisa A, Kvietkauskas M, Beisa V, Stoskus M, Ostaneviciute E, Jasiunas E, Griskevicius L, Seinin D, Sileikyte A, Strupas K 2019 Significance of BRAF V600E mutation and cytomorphological features for the optimization of papillary thyroid cancer diagnostics in cytologically indeterminate thyroid nodules. Exp Clin Endocrinol Diabetes **127:**247–254.

S130. Valderrabano P, Khazai L, Thompson ZJ, Otto KJ, Hallanger-Johnson JE, Chung CH, Centeno BA, McIver B 2018 Association of tumor size with histologic and clinical outcomes among patients with cytologically indeterminate thyroid nodules. JAMA Otolaryngol Head Neck Surg **144:**788–795.

S131. Zhang Y, Zhang Z, Ma J, Pu J, Hou P, Yang Q 2018 High-accuracy detection of preoperative thyroid nodules using combination of BRAF(V600E) mutation and TMPRSS4 mRNA level. Arch Med Res **49:**365–372.
